# Integrated multi-omics reveals the roles of cecal microbiota and its derived bacterial consortium in promoting chicken growth

**DOI:** 10.1128/msystems.00844-23

**Published:** 2023-11-29

**Authors:** Meihong Zhang, Depeng Li, Xinyue Yang, Fuxiao Wei, Qiu Wen, Yuqing Feng, Xiaolu Jin, Dan Liu, Yuming Guo, Yongfei Hu

**Affiliations:** 1 State Key Laboratory of Animal Nutrition and Feeding, College of Animal Science and Technology, China Agricultural University, Beijing, China; University of California San Diego, La Jolla, California, USA

**Keywords:** chicken, growth performance, cecal microbiota, metabolomics, transcriptomics, targeted culturomics

## Abstract

Growing evidence has shown a close connection between gut microbiota and chicken growth performance; however, the crosstalk between microbes and chicken host remains elusive. Here, we integrated multi-omics approaches, fecal microbiota transplantation, and body weight-associated bacterial consortium to investigate the host-microbiota interactions in different body weight chickens. Microbial profiling analysis showed that uncultured *Barnesiellaceae*, *Lactobacillus*, *Bacillus*, *Ruminococcaceae UCG-004*, and *Ruminococcaceae UCG-014* were highly enriched in the high body weight (HBW) chickens. The combination of *Lactobacillus* and *Bacillus* had 95.1% accuracy in discriminating HBW from low body weight chickens. Lipids and lipid-like molecules were found to be more abundant in the HBW chickens, and the differentially expressed cecal genes were enriched in the peroxisome proliferator-activated receptor (PPAR) signaling pathway and calcium signaling pathway. Correlations among the weight-associated genera, gut content metabolites, and gut gene expression were established, and fecal microbiota transplantation from HBW microbiota to newly born chicks increased the chicken antioxidant status, gut sugar transport, and immunity. A total of 67 strains belonging to *Lactobacillus* and *Bacillus* were isolated from the HBW chickens by the targeted culturomics method. Among six pairwise combinations of four selected strains, the consortium consisting of *Limosilactobacillus reuteri* CML393 and *Bacillus velezensis* CML396 significantly improved the chicken growth performance and gut health and influenced the cecal microbiota, metabolites, and gene expression. Further *in vitro* and *in silico* analyses indicated that *L. reuteri* CML393 and *B. velezensis* CML396 were less competitive but more cooperative than other pairwise combinations tested.

**IMPORTANCE:**

The improvement of chicken growth performance is one of the major concerns for the poultry industry. Gut microbes are increasingly evidenced to be associated with chicken physiology and metabolism, thereby influencing chicken growth and development. Here, through integrated multi-omics analyses, we showed that chickens from the same line differing in their body weight were very different in their gut microbiota structure and host-microbiota crosstalk; microbes in high body weight (HBW) chickens contributed to chicken growth by regulating the gut function and homeostasis. We also verified that a specific bacterial consortium consisting of isolates from the HBW chickens has the potential to be used as chicken growth promoters. These findings provide new insights into the potential links between gut microbiota and chicken phenotypes, shedding light on future manipulation of chicken gut microbiota to improve chicken growth performance.

## INTRODUCTION

A large number and wide variety of microorganisms, mainly bacteria, inhabit the chicken gastrointestinal tract, with the highest bacterial diversity found in the cecum (1). Increasing evidence has shown that the gut microbial community plays a critical role in modulating chicken physiology and metabolism, thereby contributing to the chicken growth performance, feed efficiency, nutrient absorption, host defense, and immune response (2–4).

The growth and development of chickens have always been important concerns in poultry science. Nutrition, genetics, and environments are all known to be implicated in better chicken growth performance. In recent years, more and more studies have shown that chickens with different growth performances are very different in their gut microbiota composition and diversity. For example, 32 microbial taxa are found significantly different in the jejunal content and mucosa between the high body weight (HBW) and the low body weight (LBW) chickens, and *Lactobacilli* is the predominant species in HBW chickens (5). Also, the chicken body weight is found negatively correlated with the relative abundance of bacterial taxa such as *Akkermansia* and *Streptococcus*, but positively correlated with *Bifidobacterium* and *Lactococcus* (6). In addition to these correlation analyses, manipulating the gut microbiota through fecal microbiota transplantation (FMT) and probiotics have both been verified to affect chicken growth and development. Transferring fecal microbiota from 30-day-old chickens with good feed efficiency to newly born chicks tends to increase feed intake and body weight gain in female chickens (7). Administration of fecal microbiota from chickens with high feed conversion ratios to the receivers influences early colonization of gut microbiota, intestinal permeability, gut morphology, and innate immune responses (8). Reconstitution of the gut microbiota by supplementing probiotics such as a mixture of *Lactobacillus reuteri*, *Bacillus subtilis,* and *Saccharomyces cerevisiae* has a positive effect on the plasma immunoglobulin levels and the growth performance of chickens (9). Thise evidence together indicates a close connection between gut microbiota and chicken physiology and metabolism and reveal the causal relationships between gut microbes and chicken growth and development.

Gut microbes produce a variety of metabolites from dietary components or endogenous compounds produced by the microbes and the hosts. These metabolites are the main players in mediating host-microbiota crosstalk, especially at the microbiota–mucosal interface (10–12). The change in the gut microbiota structure is always accompanied by an alteration of the gut microbial metabolites, as well as the communication between microbial metabolites and gut epithelial cells. For instance, dietary fiber promotes the growth of short-chain fatty acid (SCFA)-producers, and the resulting SCFAs, especially butyrate, provide energy for the gut epithelial cells to grow and function (13). Also, gut microbes are known to generate amino acids, vitamins, and bile acids, contributing to the host amino acid homeostasis, cell growth, development, activity, nutrient absorption, and metabolic regulation, respectively (14, 15).

With the advent of high-throughput techniques, multi-omics approaches have been widely used to study microbiota-host interactions. Coupling gut metagenomics with intestinal transcriptomics, 14 chicken microbial species are demonstrated to be associated with chicken abdominal fat-relevant traits (AFRT) and AFRT-correlated differentially expressed genes (DEGs) (16). Combining rumen metagenomics and metabolomics, together with serum metabolomics, *Prevotella* species are found to affect the dairy cow’s metabolism of amino acids, thereby contributing to milk protein yield (17). Through the integration of microbiota sequencing, metabolomics, and transcriptomics, the colitis-resistance phenotype in pigs is attributed to the modulatory effects of potentially beneficial gut microbes and their metabolites on the pig immune system (18). These studies provide an integrated perspective on mechanisms of action present in microbiota-mediated host physiological and metabolic changes.

**Fig 1:**
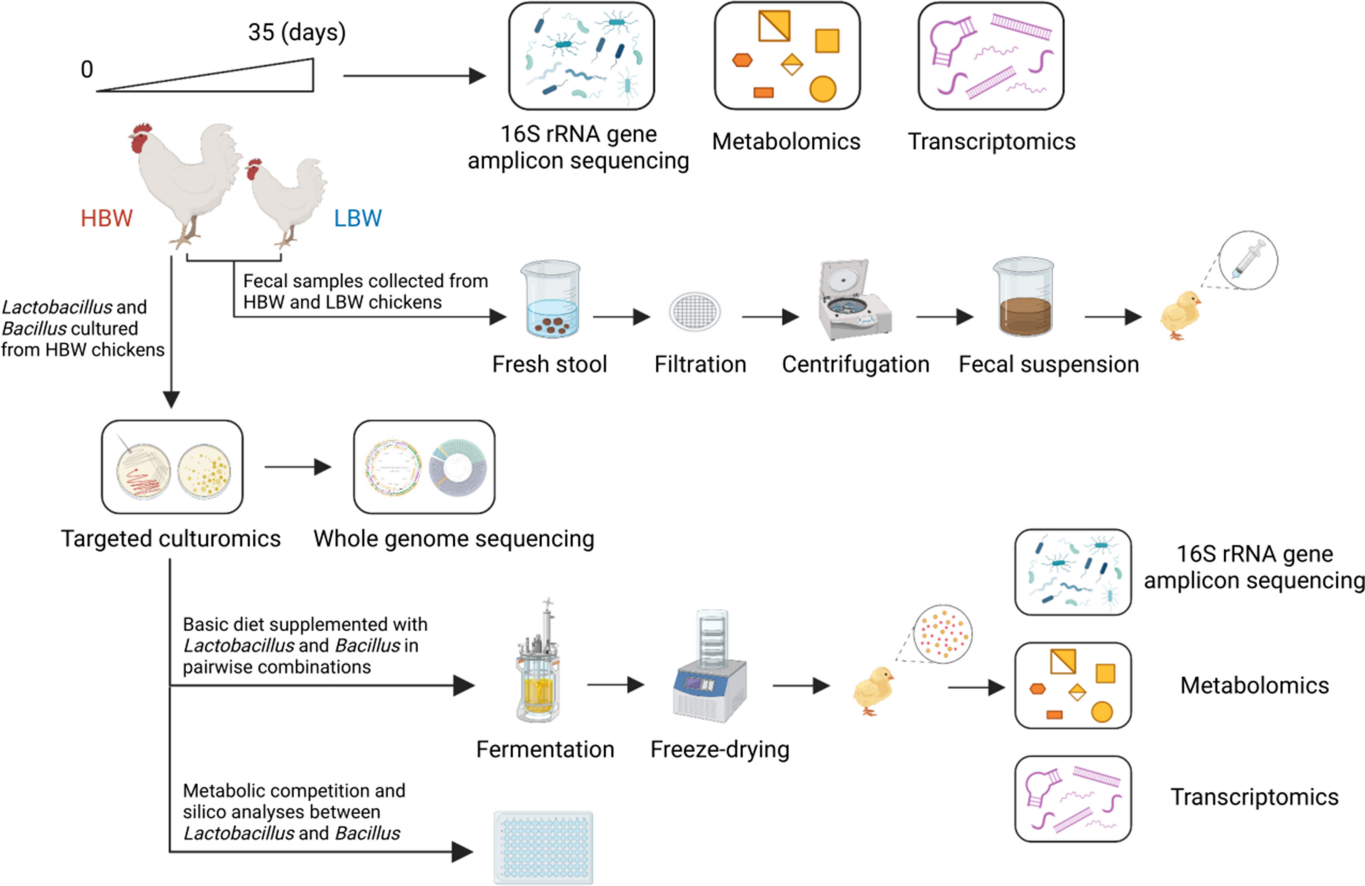
Study design for the whole experiment.

Here, we integrated multi-omics approaches including 16S ribosomal RNA (rRNA) gene amplicon sequencing, metabolomics, gut tissue transcriptomics, and targeted culturomics to investigate the host-microbiota interactions in different body weight chickens. We further explored the effects of fecal microbiota transplantation and body weight-associated bacterial consortium on improving chicken growth performance. The whole experimental design is shown in Fig. 1. The current study provides fundamental information on the host-microbiota crosstalk that promotes chicken growth performance.

## RESULTS

### The HBW and LBW chickens differ in their cecal microbiota structure and microbial interactions

**Fig 2:**
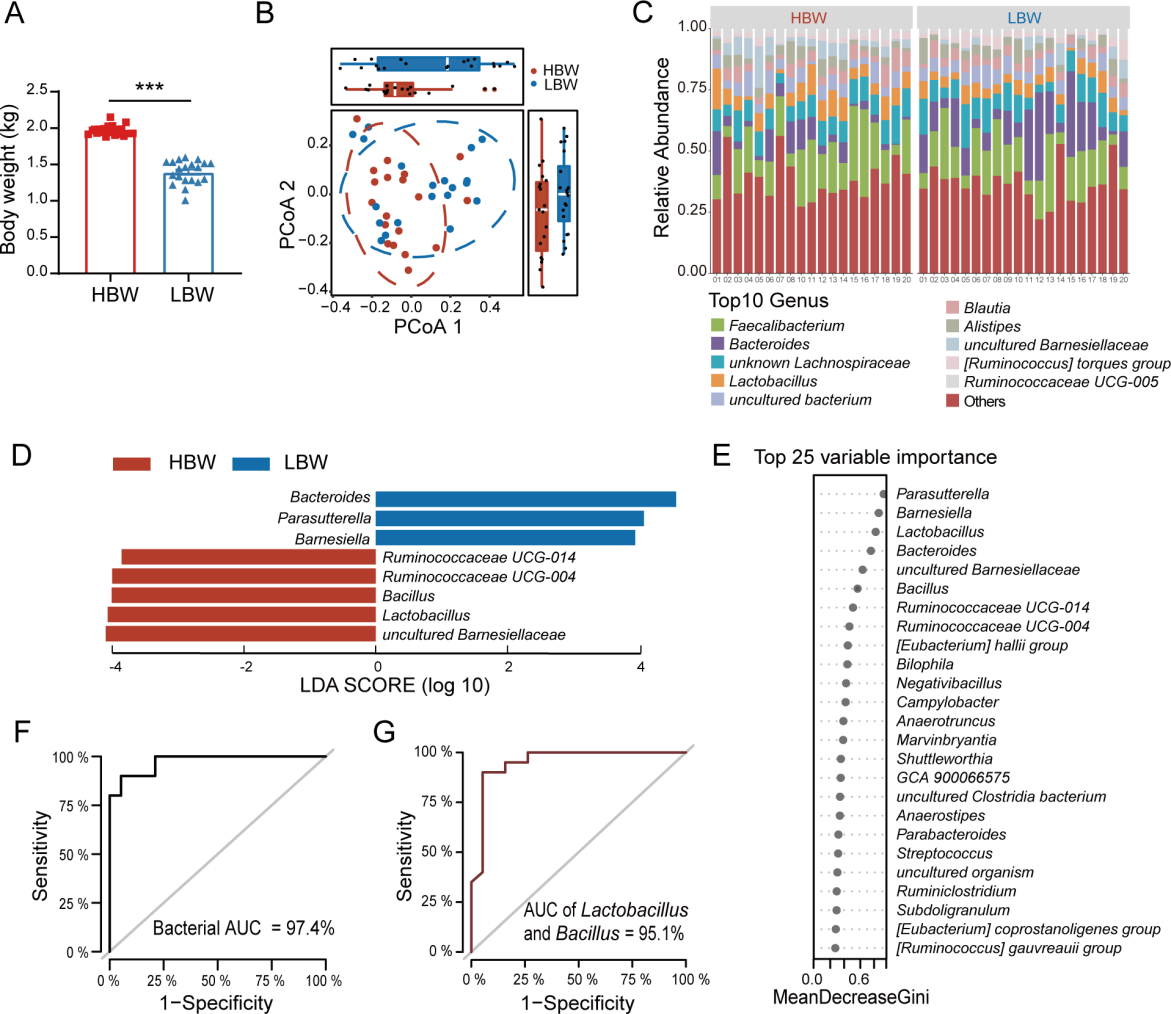
Different cecal microbiota composition between the HBW and LBW chickens. (**A**) Body weight of the HBW and LBW chickens. Data were presented as means ± SEM. Statistical significance was determined using a *t*-test. (*P-value < 0.05, **P-value < 0.01, and ***P-value < 0.001). (**B**) Principal coordinate analysis (PCoA) plot based on Bray-Curtis distance of cecal microbiota composition between the HBW and LBW chickens. Values of PCoA axis1 and PCoA axis2 were box plotted on the top and the right, respectively. (**C**) Relative abundances of cecal microbiota at the genus level in the HBW and LBW chickens. (**D**) Differentially abundant taxa were tested by linear discriminant analysis effect size, with linear discriminant analysis (LDA) score > 2 and *P*-value < 0.05. (**E**) Random forest analysis of cecal microbiota at the genus level between the HBW and LBW chickens. (**F**) ROC curve based on the top 25 marker genera for the HBW and LBW chickens. (**G**) ROC curve analysis of combinatorial taxa between *Lactobacillus* and *Bacillus*.

Based on the body weight of chickens at 35 days old, 20 HBW chickens and 20 LBW chickens were selected from 150 healthy chickens for microbiome, metabolome, and transcriptome analyses. The body weight of the HBW and LBW chickens was significantly different (mean_HBW = 1.968, , mean_LBW = 1.394, *P*-value  <  0.001; Fig. 2A).

To reveal the differences in gut microbiota composition in the HBW and LBW chickens, we performed 16S rRNA gene amplicon sequencing of the chicken ileal and cecal contents (Tables S1 and S2). The results showed that there were no significant differences in the α- and β-diversity of the ileal microbiota between the two groups of chickens (*P*-value > 0.05; Fig. S1A through E). Differentially abundant taxa were only found at the phylum level; *Firmicutes* was highly represented in the HBW group, while *Epsilonbacteraeota* was more abundant in the LBW group (Fig. S1F). We then focused on the cecal microbiota and found that no differences existed in the α-diversity (*P*-value > 0.05; Fig. S2A through D), and microbial dispersion did not change significantly (permutation multivariate dispersion [PERMDISP, *P*-value = 0.414), but Bray-Curtis distance-based β-diversity analysis showed clear separations of the two groups (permutational multivariate analysis of variance [PERMANOVA], *P*-value = 0.043; Fig. 2B). Taxonomic analysis identified 25 bacterial phyla and 397 bacterial genera in the cecal microbiota. *Firmicutes* and *Bacteroidetes* accounted for 97.14% of the total microbial community (Fig. S2E), and the *Firmicutes*/*Bacteroidetes* ratio was significantly higher in the HBW group than that in the LBW group (*P*-value = 0.001; Fig. S2F). At the genus level, 67 genera were predominant, with *Faecalibacterium* (15.80%), *Bacteroides* (9.97%), *unknown Lachnospiraceae* (9.07%), *Lactobacillus* (6.11%), *uncultured bacterium* (5.30%), and *Blautia* (4.18%) being the most abundant (Fig. 2C). The linear discriminant analysis effect size (LEfSe) analysis showed that uncultured *Barnesiellaceae*, *Lactobacillus*, *Bacillus*, *Ruminococcaceae UCG-014*, and *Ruminococcaceae UCG-004* were significantly enriched in the HBW chickens, while *Bacteroides*, *Parasutterella* and *Barnesiella* were more abundant in the LBW chickens (Fig. 2D).

To find whether there were marker bacteria to discriminate HBW chickens from LBW chickens, random forest (RF) analysis based on the genus abundance was performed. Five repeats of 10-fold cross-validation using the data set (*n* = 19 and 20 for HBW and LBW, respectively) led to the optimal selection of 25 genus markers, *Parasutterella*, *Barnesiella*, *Lactobacillus*, *Bacteroides*, uncultured *Barnesiellaceae*, *Bacillus*, *Ruminococcaceae UCG-014*, *Ruminococcaceae UCG-004*, (*Eubacterium*) *hallii* group, *Bilophila*, *Negativibacillus*, *Campylobacter*, *Anaerotruncus*, *Marvinbryantia*, *Shuttleworthia*, *GCA 900066575*, uncultured *Clostridia bacterium*, *Anaerostipes*, *Parabacteroides*, *Streptococcus*, uncultured organism, *Ruminiclostridium*, *Subdoligranulum*, (*Eubacterium*) *coprostanoligenes* group, and (*Ruminococcus*) *gauvreauii* group. The area under the curve (AUC) of the receiver operating characteristic (ROC) curve for the RF data set using 25 marker genera identified reached 97.4% (Fig. 2E and F), indicating that the RF model had good power to distinguish the two chicken groups. Of note, variables with the top eight highest variable importance in the RF model were exactly the genera that were differentially abundant between the HBW and LBW chickens, and when *Lactobacillus* and *Bacillus* were combined, the accuracy of discrimination reached 95.1% (Fig. 2G). We then investigated the cecal microbial co-occurrence networks in the two groups. The co-occurrence analysis based on the relative abundance of the genus showed that compared with the HBW group, the network of the LBW group had a higher degree and clustering coefficient (Table S3). The results suggested that the bacterial network of the LBW group was more complex than that of the HBW group.

Collectively, these results showed that the HBW and LBW chickens had different cecal microbiota structure, and the differentially abundant microbes, especially those from *Lactobacillus* and *Bacillus* in the two groups, can be used as marker bacteria to discriminate HBW chickens from LBW chickens.

### Chickens with different body weights have distinct cecal microbial metabolite signatures

**Fig 3:**
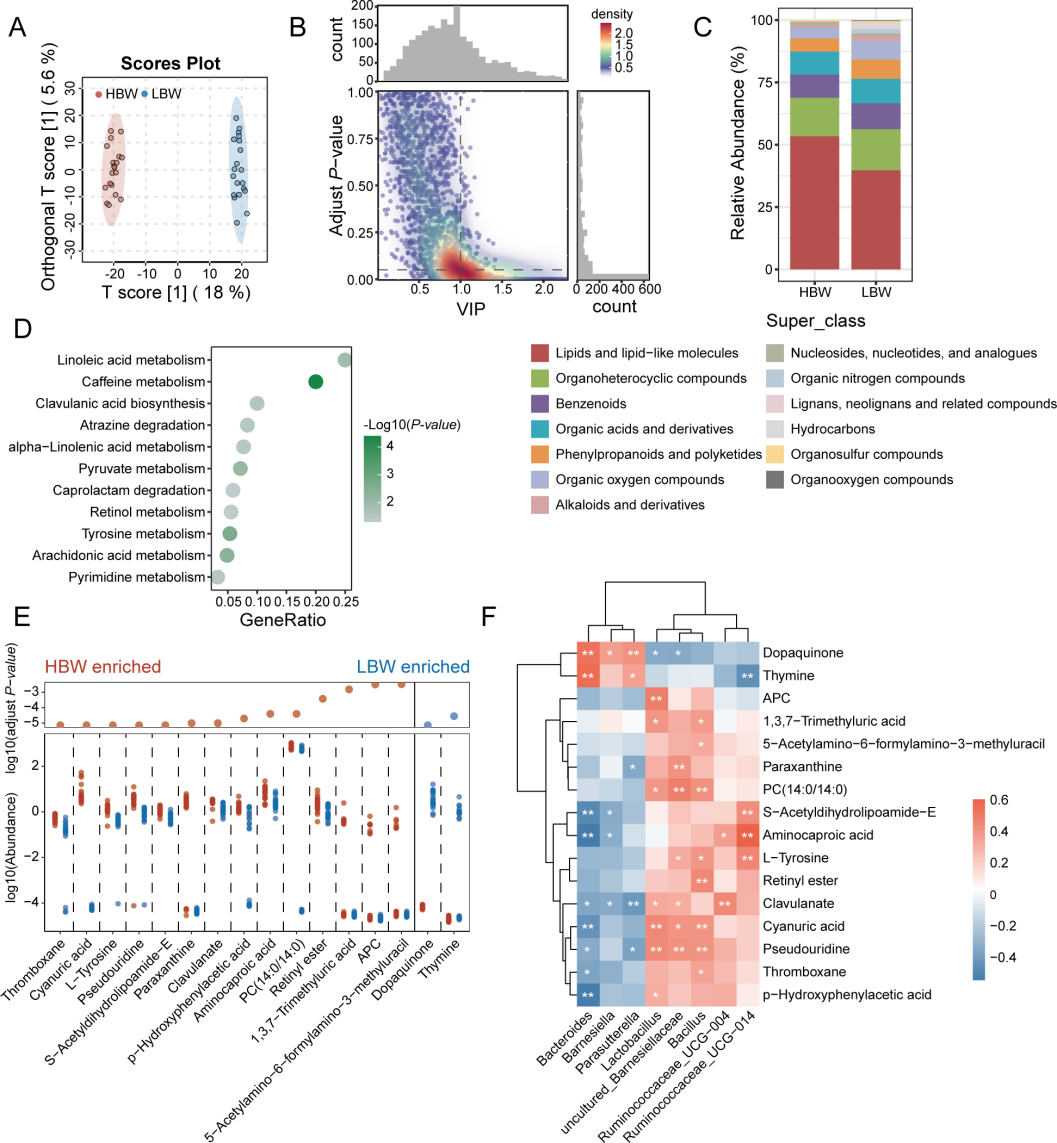
Changes in the cecal metabolite profiles of the HBW and LBW chickens. (**A**) OPLS-DA of cecal metabolites between the HBW (red) and LBW (blue) chickens. Intergroup variation is reflected by the *T*-score, and intragroup variations are reflected by orthogonal the *T*-score. (**B**) SCMs in the HBW and LBW chickens. Top panel: density of the VIP of the metabolites and right panel: density of the adjusted *P-*value. (**C**) Classification of SCMs at the superclass level in the HBW and LBW chickens. (**D**) KEGG enrichment analysis was performed using the SCMs between the HBW and LBW chickens (*P*-value < 0.05). (**E**) The abundance of the key SCMs in the HBW and LBW chickens. (**F**) Clustered heatmaps show the correlations between differentially abundant bacterial genera and the key SCMs. Significant correlations were picked with *P*-value < 0.05 (*P-value < 0.05 and **P-value < 0.01). The color scale bar indicates the correlation coefficient (*r* value).

We performed untargeted metabolomics to further investigate the changes in cecal content metabolites between the HBW and LBW chickens. A total of 2,185 metabolites were annotated from the two groups of chickens. Among them, 1,167 metabolites were found in positive ion mode, while 1,018 metabolites were found in negative ion mode. The orthogonal partial least square discriminant analysis (OPLS-DA) showed clear separations of the two groups, and the intragroup variations of the LBW chickens were higher than the HBW chickens (Fig. 3A). A total of 621 significantly changed metabolites (SCMs) between the HBW and LBW chickens were identified under the criteria of variable importance for the projection (VIP) > 1 and adjusted *P*-value < 0.05 (Wilcoxon rank-sum test), among which 324 displayed higher abundance in HBW chickens, while 297 were enriched in LBW chickens (Fig. 3B; Table S4). SCMs highly represented in the HBW chickens were classed into nine metabolic superclasses according to HMDB, among which 173 metabolites (53.40%) belonged to lipids and lipid-like molecules. In contrast, SCMs enriched in the LBW chickens belonged to 13 categories, but only 118 metabolites (39.73%) were affiliated to the superclass of lipids and lipid-like molecules (Fig. 3C), suggesting that the cecal microbes were more related to lipids and lipid-like molecules in HBW chickens than that in LBW chickens. Kyoto Encyclopedia of Genes and Genomes (KEGG) enrichment analysis based on these SCMs revealed that linoleic acid metabolism, alpha-linolenic acid metabolism, and arachidonic acid metabolism belonging to lipid metabolism were the significantly different pathways (*P*-value < 0.05; Fig. 3D; Table S5). Of the key SCMs related to lipid metabolic pathways, thromboxane and phosphatidylcholine (PC)(14:0/14:0) were present at a higher abundance in the HBW chickens (*P*-value < 0.05; Fig. 3E).

To explore the relationships between the chicken cecal microbes and the gut content metabolites, correlation analyses were performed. First, the eight differentially abundant bacterial genera between the two groups were found to be significantly correlated with 16 key SCMs (Fig. 3F). The HBW-enriched uncultured *Barnesiellaceae*, *Lactobacillus*, *Bacillus*, *Ruminococcaceae UCG-014*, and *Ruminococcaceae UCG-004* had more positive correlations than negative correlations (26 vs 3) with key metabolites, while LBW-enriched *Bacteroides*, *Parasutterella*, and *Barnesiella* showed more negative than positive correlations (13 vs 5). Strikingly, *Lactobacillus* and *Bacillus* were positively correlated with the thromboxane and PC(14:0/14:0), suggesting their potential role in modulating lipid metabolism.

### The cecal transcriptomic profiles are different between the HBW and LBW chickens

**Fig 4:**
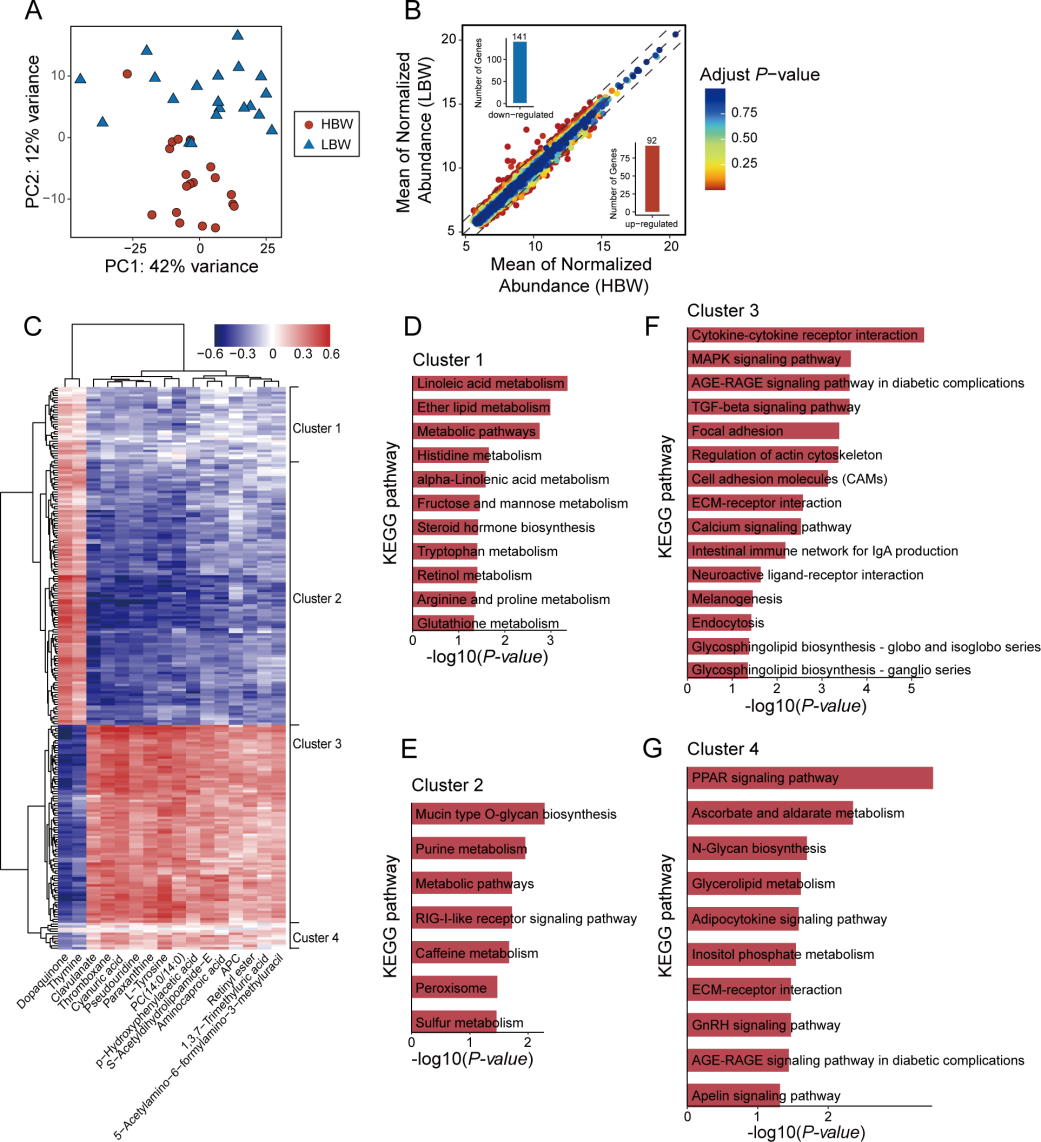
Cecal tissue transcriptomic profiles of the HBW and LBW chickens. (**A**) PCA plots of expression levels from all genes between the HBW and LBW chickens. (**B**) DEGs in the HBW and LBW chickens. The upregulated (*n* = 92) and downregulated genes (*n* = 141) in HBW chickens are shown in red (bottom right) and blue (top left), respectively. (**C**) Clustered heatmaps show the correlations between the key SCMs and DEGs. Significant correlations were picked with *P*-value < 0.05. The color scale bar indicates the correlation coefficient (*r* value). KEGG enrichment analysis was performed using the DEGs in cluster 1 (**D**), cluster 2 (**E**), cluster 3 (**F**), and cluster 4 (**G**) between the HBW and LBW chickens (*P*-value < 0.05).

To reveal the differences in cecal gene expression between the two groups of chickens, RNA-seq analysis was performed on cecum tissue samples. After stringent quality filtering of the raw reads, 29,122,451 clean reads on average were obtained per sample, and more than 80% of the reads had a quality score of Q30. These clean reads were mapped to the chicken reference genome with relatively higher efficiency (65%–92% in different samples) (Table S6). Principal component analysis (PCA) indicated that the cecal gene expression profiles in the two groups of chicken were very different: samples from different groups were separately clustered and samples from the same group were closer to each other (Fig. 4A). A total of 233 genes were found differentially expressed in these chickens, with 92 genes upregulated and 141 genes downregulated in HBW chickens compared with LBW chickens (Fig. 4B; Table S7).

A further correlation analysis between the gut content metabolites and host gene expression showed that a total of 2,318 correlations were found, among which 1,094 were positive and 1,224 were negative correlations (*P*-value < 0.05; Fig. 4C). According to the heatmap clustering, the SCMs-associated DEGs were grouped into four major clusters. Cluster 1 (30 DEGs) was more involved in ether lipid metabolism (*P*-value < 0.05; Fig. 4D), and cluster 2 (110 DEGs) was mostly associated with mucin-type O-glycan biosynthesis and purine metabolism (*P*-value < 0.05; Fig. 4E). Cluster 3 contained 82 DEGs mainly enriched in cytokine-cytokine receptor interaction, MAPK signaling pathway, and calcium signaling pathway (*P*-value < 0.05; Fig. 4F). Cluster 4 contained 11 DEGs closely related to PPAR signaling pathway and glycerolipid metabolism (*P*-value < 0.05; Fig. 4G). In particular, we found that the HBW-enriched thromboxane and PC(14:0/14:0) were positively associated with the changes of DEGs in Clusters 3 and 4, and negatively correlated with Clusters 1 and 2 (Fig. 4C). Together, these results suggest that microbes in the chicken cecum may affect the gut function through various gut content metabolites.

### FMT enhances chicken antioxidant capacity, gut sugar transport, and immunity

**Fig 5:**
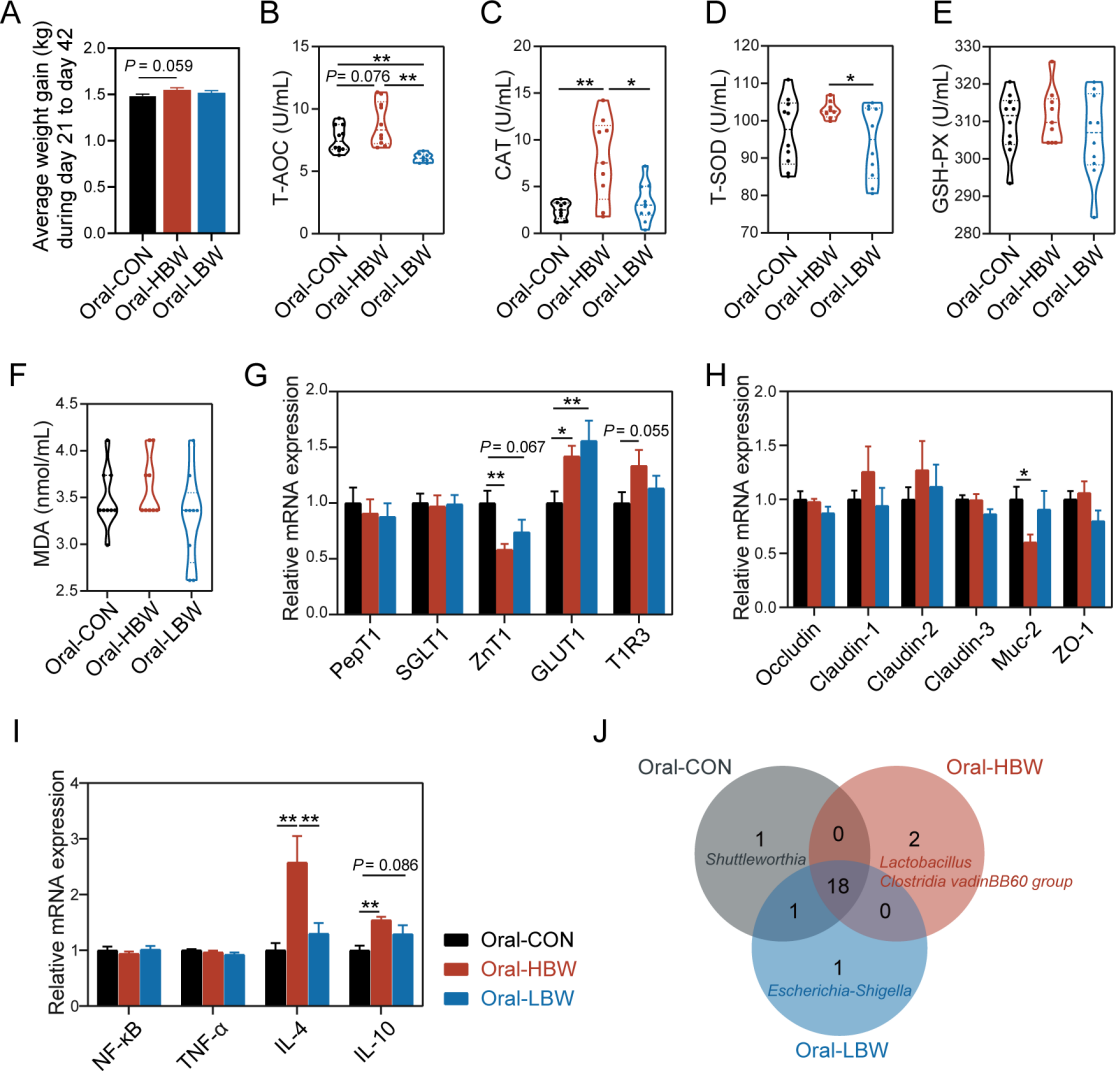
Effects of FMT on growth performance, serum antioxidant, relative mRNA expression, and cecal microbiota of chickens. (**A**) Average weight gain from day 21 to day 42. The concentrations of T-AOC (**B**), CAT (**C**), T-SOD (**D**), GSH-PX (**E**), and MDA (**F**) in the serum of chickens fed with different fecal suspensions. (**G**) The relative mRNA expression of sugar transporter proteins in the jejunum. (**H**) The relative mRNA expression of tight junction proteins in the jejunum. (**I**) The relative mRNA expression of immunity-related genes in the jejunum (*P-value < 0.05, **P-value < 0.01, and ***P-value < 0.001). (**J**) Venn diagram of core microbiome membership among the Oral-CON, Oral-HBW, and Oral-LBW groups.

Next, we investigate the effects of the gut microbiota from HBW and LBW chickens on the growth performance of newly born chicks by FMT. The results showed that microbiota from HBW and LBW chickens had no observable effects on the growth performance of chickens (*P*-value > 0.05; Fig. S3); the Oral-HBW group only displayed a slightly increased average weight gain from day 21 to day 42 compared with the Oral-CON group (*P*-value = 0.059; Fig. 5A). However, microbiota from HBW chickens tended to increase the receivers’ antioxidant activity. Compared with the Oral-CON group, the total antioxidant capacity (T-AOC) tended to be increased by microbiota from HBW chickens (*P*-value = 0.076) but was significantly decreased by microbiota from LBW chickens (*P*-value < 0.01; Fig. 5B). Similarly, the catalase (CAT) activity in chickens treated with HBW microbiota was remarkably increased compared with those treated with sterile saline and LBW microbiota (*P*-value < 0.05; Fig. 5C). The concentration of total superoxide dismutase (T-SOD) was not affected by HBW or LBW microbiota compared with the Oral-CON group, but higher concentration was found in the Oral-HBW chickens than in the Oral-LBW chickens (*P*-value < 0.05; Fig. 5D), while microbiota from either HBW or LBW chickens had no effects on the concentrations of glutathione peroxidase (GSH-PX) and malondialdehyde (MDA) (*P*-value > 0.05; Fig. 5E and F).

We also measured the expression of genes related to sugar transporter, tight junction proteins, and inflammation in the FMT receivers. Compared with Oral-CON group, microbiota from HBW chickens significantly reduced the relative mRNA expression of zinc transporter 1 (*P*-value < 0.01) but markedly increased the mRNA expression of glucose transporter type 1 (*P*-value < 0.05), and an increasing trend was observed for the expression of taste receptor family 1 member 3 (*P*-value = 0.055; Fig. 5G). FMT had little effect on the expression levels of the tight junction proteins, but microbiota from HBW chickens reduced the relative mRNA expression of mucin 2 (MUC-2) compared with Oral-CON (*P*-value < 0.05; Fig. 5H). Besides, the relative mRNA expression of anti-inflammatory cytokines such as interleukin 4 and interleukin 10 in the chicken jejunum was significantly increased after receiving HBW microbiota (*P*-value < 0.01; Fig. 5I).

To further investigate the effect of FMT on the gut microbiome, the flora of the cecum was assessed using 16S rRNA gene amplicon sequencing. Microbial profiling analysis revealed that FMT had no significant effect on the microbial community structure (Fig. S4A), but slightly changed core microbiome membership (Fig. S4B through D). The Venn diagram (Fig. 5J) showed that 18 microorganisms were shared among the oral-CON, oral-HBW, and oral-LBW groups, revealing the presence of a strong core microbiota. Moreover, *Shuttleworthia* and *Escherichia-Shigella* were unique core microbiome membership in the oral-CON and oral-LBW groups, respectively. Of note, HBW-enriched *Lactobacillus* became the core microbiome member of the oral-HBW group after transplanting the feces of HBW chickens.

These results indicated that FMT from HBW microbiota to newly born chicks slightly affected chicken growth performance and gut core microbiome membership, but seemed to enhance the ability of antioxidant status, sugar transport, and immunity.

### A bacterial consortium consisting of *Limosilactobacillus reuteri* and *Bacillus velezensis* isolated from the HBW chickens improves chicken growth performance

As we showed above, the highly represented *Lactobacillus* and *Bacillus* in the HBW chickens were probably key taxa in shaping the community structure (Fig. 1D and G), we wondered if combinations of strains from these two genera from the HBW chickens instead of the whole microbiota had the growth-promoting effect. We first performed targeted culturomics to isolate *Lactobacillus* and *Bacillus* species from the HBW chickens. A total of 95 bacterial isolates were obtained and identified by the 16S rRNA gene (Table S8). These isolates mainly belonged to six *Lactobacillus* species and six *Bacillus* species. *Ligilactobacillus salivarius* CML391, *Limosilactobacillus reuteri* CML393, *Bacillus velezensis* CML396, and *Bacillus paralicheniformis* CML399 with high frequencies of isolation were selected for the subsequent analyses. We sequenced the whole genomes of these four strains (Table S9), and the whole genome SNPs-based phylogenetic analysis indicated that *L. Salivarius* CML391 and *L. reuteri* CML393 were relatively far from other isolates that have been sequenced, while *B. velezensis* CML396 and *B. paralicheniformis* CML399 were closest to *B. velezensis* QC-J and *B. paralicheniformis* CBMAI 1303, respectively (Fig. S5).

**Fig 6:**
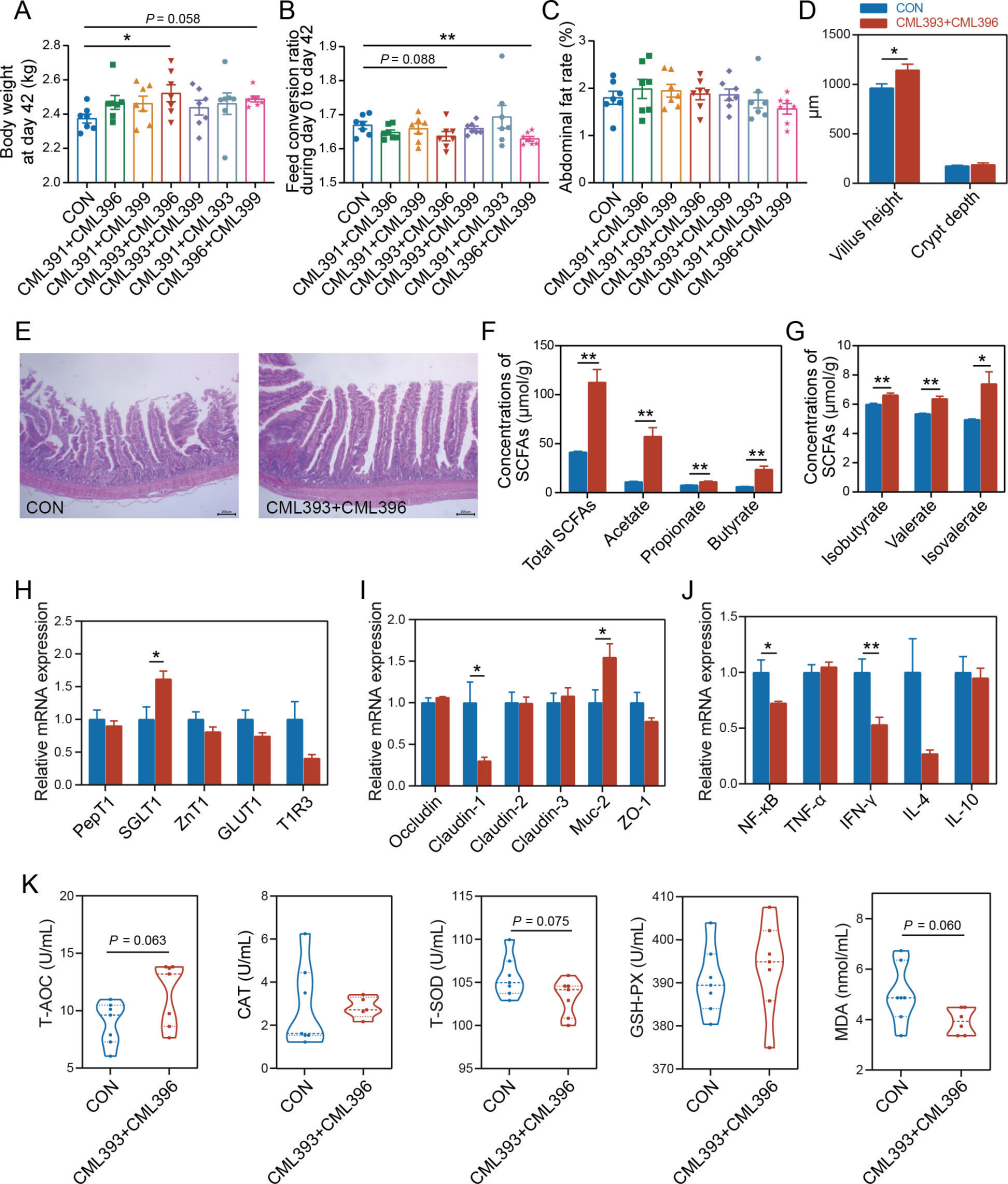
Effects of dietary supplementation with *L. reuteri* CML393 and *B. velezensis* CML396 on chicken growth performance and gut health. (**A**) Body weight on the 42nd day. (**B**) Feed conversion ratio from day 0 to day 42. (**C**) Abdominal fat rate on the 42nd day. (**D**) The difference in jejunum villus height and crypt depth between CON and CML393 + CML396 chickens. (**E**) Jejunum epithelial morphology of CON and CML393 + CML396 chickens. Scale bars = 200 µm. (**F**) Concentrations of major SCFAs in the cecum of CON and CML393 + CML396 chickens. (**G**) Concentrations of minor SCFAs in the cecum of CON and CML393 + CML396 chickens. (**H**) The relative mRNA expression of sugar transporter proteins in the jejunum. (**I**) The relative mRNA expression of tight junction proteins in the jejunum. (**J**) The relative mRNA expression of immunity-related genes in the jejunum. (**K**) Effects of *L. reuteri* CML393 and *B. velezensis* CML396 supplementation on serum antioxidant of chickens (*P-value < 0.05 and ** *P*-value < 0.01).

We then evaluated the growth-promoting effects of the pair-wise combinations of the four strains (six consortiums) on newly born chicks. Compared with the control group, the six consortiums had no obvious effect (*P*-value > 0.05) on the growth performance of chickens from day 0 to day 21 (Fig. S6A through D). Between 21 and 42 days of age, there was a dramatic increase in body weight of chickens fed with the consortium consisting of *L. reuteri* CML393 and *B. velezensis* CML396 (*P*-value < 0.05; Fig. 6A; Fig. S6E through I). Meanwhile, this two-strain combination tended to decrease the feed conversion ratio from day 0 to day 42 (*P*-value = 0.088; Fig. 6B); and there was no significant difference in abdominal fat rate between the two-strain combination (*P*-value > 0.05; Fig. 6C).

We next analyzed the effect of this consortium on chicken gut health and found that it significantly increased the jejunum villus height (*P*-value < 0.05; Fig. 6D and E), and the concentrations of total SCFAs, acetate, propionate, butyrate, isobutyrate, valerate, and isovalerate in the chicken cecum (*P*-value < 0.05; Fig. 6F and G). Moreover, this consortium remarkably increased the relative mRNA expression of sodium-glucose cotransporter 1 and MUC-2 and decreased the relative mRNA expression of claudin-1 (*P*-value < 0.05), while having no obvious effect on other tight junction protein-related genes, i.e., zonula occludens-1, occludin, claudin-2, and claudin-3 (*P*-value > 0.05; Fig. 6H and I). Additionally, the combination of *L. reuteri* CML393 and *B. velezensis* CML396 significantly reduced the relative mRNA expression of nuclear factor kappa-B and interferon-gamma (*P*-value < 0.05; Fig. 6J) but did not improve the serum antioxidant activities (*P*-value > 0.05; Fig. 6K).

Collectively, these results suggest that the specific combination of *Lactobacillus* and *Bacillus* isolates enriched in the HBW chickens has the ability to promote chicken growth performance and to a certain degree improve chicken gut health.

### *L*. *reuteri* CML393 and *B*. *velezensis* CML396 combination affects the cecal microbiota, metabolites, and gene expression

**Fig 7:**
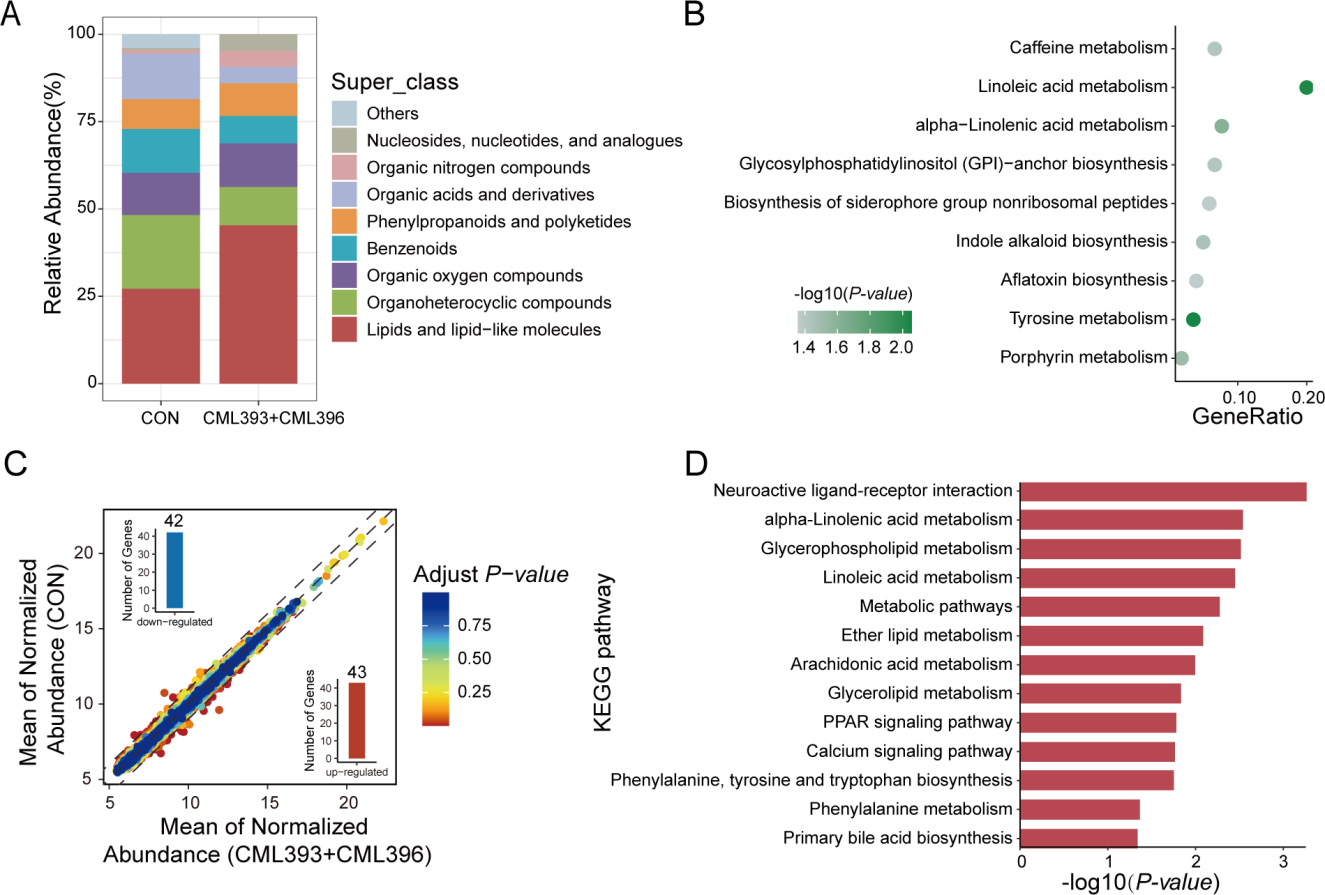
Effects of dietary supplementation with *L. reuteri* CML393 and *B. velezensis* CML396 on cecal metabolites and gene expression of chickens. (**A**) Classification of SCMs at the superclass level in the CON and CML393 + CML396 chickens. (**B**) KEGG enrichment analysis was performed using the SCMs between the CON and CML393 + CML396 chickens (*P*-value < 0.05). (**C**) DEGs in the CON and CML393 + CML396 chickens. Compared with the CON group, the 43 upregulated genes are shown in red (bottom right) and 42 downregulated genes in blue (top left). (**D**) KEGG enrichment analysis of DEGs between the CON and CML393 + CML396 chickens (*P*-value < 0.05).

To reveal the mechanisms underlying the growth-promoting effects of the *L. reuteri* CML393 and *B. velezensis* CML396 combination and to compare with that in the HBW chickens, again we investigated the alterations of chicken cecal microbiota, metabolites, and gene expression after the intervention of this bacterial consortium. Dietary combination of *L. reuteri* CML393 and *B. velezensis* CML396 did not affect either the cecal microbial α- or β-diversity (Fig. S7A through C). However, co-occurrence network analysis showed less complex bacterial interactions in the CML393 + CML396 group compared with the control group (Table S10), which was consistent with the comparison between the HBW and LBW chickens. The metabolomic analysis identified a total of 263 SCMs (Fig. S7D; Table S11), among which 64 SCMs enriched in the CML393 + CML396 group were assigned to eight metabolic superclasses, and lipids and lipid-like molecules accounted for the majority (45.31%) of the enriched SCMs (Fig. 7A). A further KEGG enrichment analysis showed that the SCMs were closely related to linoleic acid metabolism and alpha-linolenic acid metabolism (*P*-value  <  0.05; Fig. 7B). RNA-seq analysis of the cecal gene expression identified 85 DEGs including 43 upregulated genes and 42 downregulated genes in CML393 + CML396 chickens compared with the control group (Fig. 7C; Table S12). KEGG enrichment analysis further confirmed that the DEGs were significantly enriched to the PPAR signaling pathway and calcium signaling pathway (*P*-value  <  0.05; Fig. 7D), which was also found in the HBW and LBW chickens. Of note, lipids and lipid-like molecules [PC(14:0/14:0) and 7(14)-bisabolene-2,3,10,11-tetrol] and cecal genes, including pyruvate dehydrogenase kinase isozyme 4 (PDK4), transmembrane and immunoglobulin domain containing 1, and SLIT and NTRK like family member 3 (SLITRK3), found in the comparison of HBW and LBW chickens were also observed to be differentially expressed between the CML393 + CML396 and the control groups.

### *L*. *reuteri* CML393 and *B*. *velezensis* CML396 are less competitive but more cooperative

**Fig 8:**
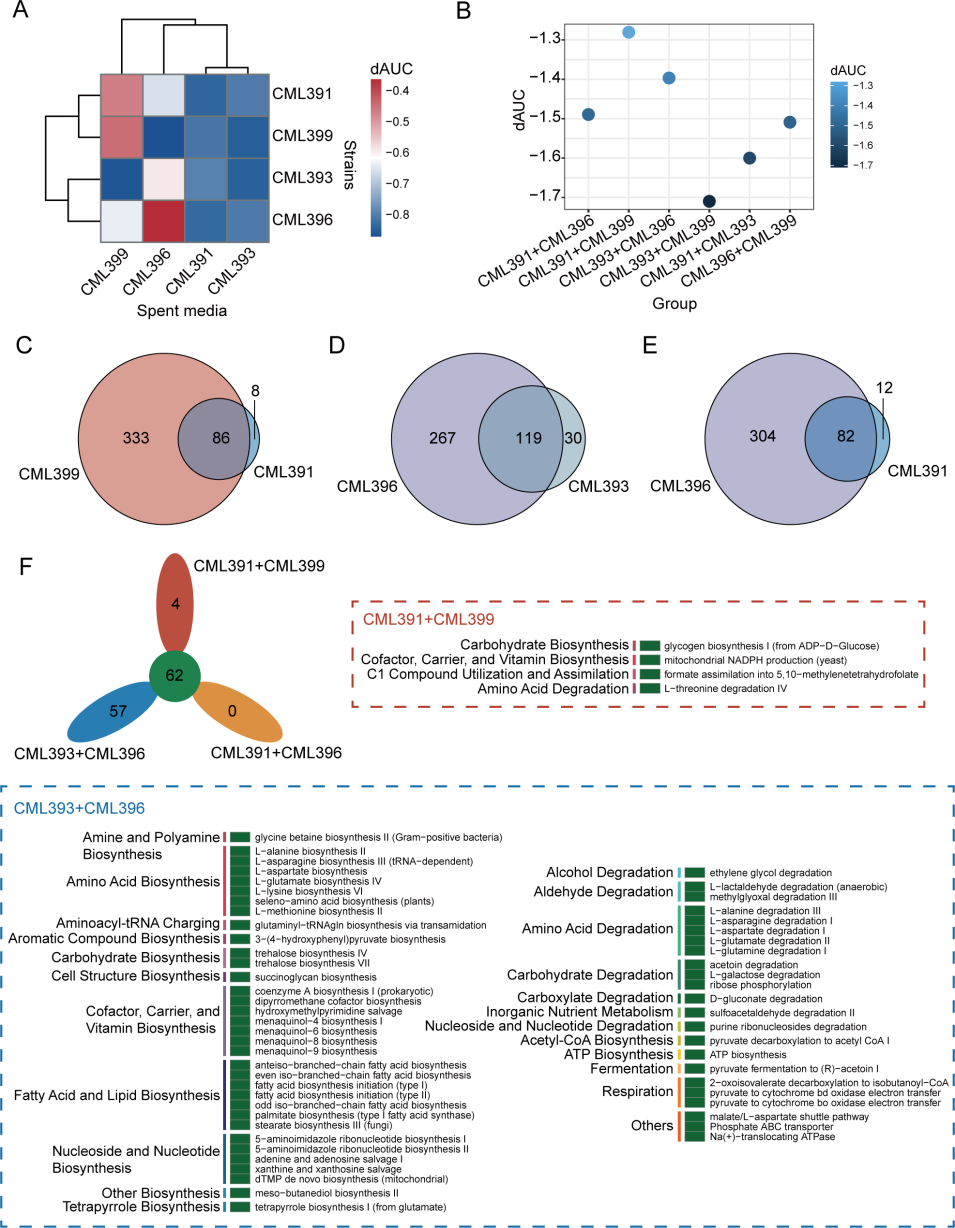
Competitive analysis and metabolic properties of the four strains in pair-wise combinations. (**A**) Monoculture growth in SM resulted in mostly decreased AUC values in comparison to fresh MIX medium, which was analyzed by calculating the inhibition factor *d*_AUC_. *d*_AUC_ = (AUC_SM_ − AUC_MIX_)/AUC_MIX_. (**B**) The *d*_AUC_ of the four strains in pair-wise combinations. (**C**) Venn diagram of the metabolic pathway of CML391 and CML399. (**D**) Venn diagram of the metabolic pathway of CML393 and CML396. (**E**) Venn diagram of the metabolic pathway of CML391 and CML396. (**F**) Common and unique metabolic pathways among different combinations. Green rectangles mean the presence of the pathway; the pathway categories were ordered by consensus functional classification.

To further investigate why the combination of *L. reuteri* CML393 and *B. velezensis* CML396 but no other consortiums exert the most obvious growth-promoting effect on the chickens, the bacterial interactions between members within each consortium were analyzed in *in vitro* assays according to previously described methods (19). First, we determined that an MIX medium (De Man Rogosa Sharpe (MRS) + Luria-Bertani (LB)) supported the growth of all four strains. Then, each strain was cultured in the sterile spent culture medium (SM) of the other strains and its own SM, and the area under the growth curve was determined over 48 hours (Fig. S8). Here, we introduced normalized inhibition factor (*d*_AUC_), which was determined by the AUC in SM relative to the AUC in fresh MIX medium [*d*_AUC_ = (AUC_SM_ − AUC_MIX_)/AUC_MIX_], to quantify the influence of different SMs on the growth of the individual strains (Fig. 8A) (19). The results showed that the SM of CML391 or CML393 reduced the growth of all strains (*d*_AUC_ <−0.7), while the SM of CML396 or CML399 just inhibited the growth of one strain (CML399 and CML393, respectively). When the two *d*_AUC_ values in each pair of strains were added together, strains in combinations of CML391 + CML399, CML393 + CML396, and CML391 + CML396 showed relatively less reciprocal inhibitory effects than strains in the other three combinations (*d*_AUC_ > −1.5; Fig. 8B).

To gain insights into the metabolic properties of these three optimal combinations, we predicted the metabolic potentials for each individual strain using gapseq. The results showed that CML393 and CML396 shared the highest number of metabolic pathways (119 pathways), followed by CML391 and CML399 (86 pathways), and CML391 and CML396 (82 pathways) (Fig. 8C through E). Among all the shared metabolic pathways in the three combinations, 62 pathways were common, and interestingly, 57 pathways were unique in CML393 + CML396 combination, while only four were unique in the CML391 + CML399 combination (Fig. 8F). The unique pathways in the CML393 + CML396 combination mainly included amino acid biosynthesis (seven pathways), fatty acid and lipid biosynthesis (seven pathways), and cofactor, carrier, and vitamin biosynthesis (seven pathways). Taken together, these results suggest that strains in the consortium of *L. reuteri* CML393 and *B. velezensis* CML396 are less competitive or antagonistic, but more cooperative in enhancing microbial beneficial metabolites.

## DISCUSSION

In this study, we revealed the role of gut microbiota in chicken growth performance by integrating 16S rRNA gene amplicon sequencing, metabolomics, and transcriptomics. Also, we demonstrated for the first time that a two-strain consortium of *L. reuteri* CML393 and *B. velezensis* CML396 from the HBW chickens, instead of the whole gut microbiota, conferred a growth advantage for chickens receiving it in the diet.

Studies have shown that the composition and diversity of gut microbiota differ between chickens with different growth performances, suggesting strong correlations between gut microbiota and growth performance (5, 6). In the present study, more differences in microbial community structure were found in the chicken cecum than in the ileum between chickens with different body weights. This was consistent with previous findings that the chicken ileal microbiota was relatively stable and probably shared a core microbiota regardless of different dietary interventions (20, 21). Moreover, the chicken’s small intestinal microbiota was found to be less influenced by the host genetics (22). The reason for this may be partially attributed to the short retention time of digesta in the gastrointestinal tract of chickens, usually less than 3.5 hours (23). However, the passage rate is slow in the caeca, a site hosting the most diverse and complex microbiota passed from the foregut that may have a more considerable effect on chicken physiology and metabolism (24). Nonetheless, our results do not mean that microbes in the chicken small intestine have no contributions to the chicken growth performance; even though some microbes are just passed through the small intestine, they may still influence chicken growth and development.

We found that the cecal microbial community structures were very different between the HBW and LBW chickens, and a significantly higher *Firmicutes*/*Bacteroidetes* ratio was observed in the HBW group. The increased *Firmicutes*/*Bacteroidetes* ratio in the HBW chickens may imply that microbes in these birds are more efficient in extracting energy from the diet and responsible for the subsequent weight gain, as it is evidenced that *Firmicutes* are more effective in promoting energy uptake than *Bacteroidetes* (25). The elevation of this ratio in the HBW chickens was further reflected by the increased abundance of *uncultured Barnesiellaceae*, *Lactobacillus*, *Bacillus*, *Ruminococcaceae UCG-014*, and *Ruminococcaceae UCG-004* and decreases in the abundance of *Bacteroides*, *Parasutterella*, and *Barnesiella*. Specific species from *Lactobacillus* (26, 27) and *Bacillus* (28, 29) are probiotics that have been demonstrated to be beneficial for gut physiology, intestinal homeostasis, and growth performance of chickens. *Ruminococcaceae*, a member of the SCFAs producers, has been implicated in the fermentation of diverse polysaccharides and fibers (30, 31), thus protecting intestinal barrier function and exerting anti-inflammatory effects in the host (32). Conversely, the LBW-enriched microbes may have harmful effects on the birds and affect their growth performance. For example, some species of *Bacteroides* are known to produce endotoxin and induce carcinogenesis via increasing the interleukin 22 expression levels or changing the bile acid metabolic pathway (33). However, there were also studies stating that *Bacteroides* belonged to propionate producers and utilized both plant and host-derived polysaccharides (34). Thus, the exact role of *Bacteroides* was still open for debate. Also, a higher abundance of *Parasutterella* was associated with irritable bowel syndrome and chronic intestinal inflammation (35, 36). Another interesting finding related to the community structure in this study is that the microbial interaction networks in the HBW and CML393 + CML396 chickens were less complex than their respective controls with worse growth performance. A very recent study suggested that more complex interactions among species in a community may reduce the community’s stability and destabilize certain ecological functions (37, 38). Our results coupled with this new finding prompt us to propose that strategies aiming at reducing the complexity of chicken gut microbiota may be beneficial for improving animal growth and development.

It is known that diverse metabolites can be produced by gut microbiota, which play important roles in mediating the complex interactions between gut microbiota and host physiology (39). In this work, we found that metabolites of lipids and lipid-like molecules displayed the most differences between the HBW and LBW chickens, suggesting the different abilities of gut microbes in processing lipids in the two groups. Of note, we also found that linoleic acid metabolism and alpha-linolenic acid metabolism belonging to lipid metabolism played important roles in both the HBW and the CML393 + CML396-supplemented chickens, further indicating a possible connection of lipids and chicken growth. PC(14:0/14:0), an important component of linoleic acid metabolism and alpha-linolenic acid metabolism, was found to be commonly enriched in chickens with better growth performance. The functions of PC(14:0/14:0) have not yet been reported, but the roles of fatty acids composed of PC(14:0/14:0), for instance, myristic acid, have been revealed. Myristic acid was found to have anti-bacterial and anti-inflammatory effects (40), and dietary fat rich in myristic acid was beneficial for increasing feed efficiency and breast muscle rate of broiler chickens (41). Together, a high representation of these lipid-associated metabolites in better-performed chickens in this study may imply their important roles in regulating chicken physiology and metabolism and shed new light on mechanisms underlying the gut microbes-mediated chicken growth and development.

Our transcriptomic results showed that the DEGs related to the PPAR signaling pathway and calcium signaling pathway in the HBW and LBW chickens were also found to be affected by the intervention of *L. reuteri* CML393 and *B. velezensis* CML396. The PPAR signaling pathway is critical in the regulation of gut physiology, including sensing nutrients (fatty acids and their derivatives), metabolizing lipids, amino acids, carbohydrates, and modulating the immune system and inflammatory response (42). Additionally, the PPARα signaling is evidenced to be necessary for enlarging the intestinal surface by increasing villi length, and for nutrient uptake by enterocytes (43). We also found that the consortium of *L. reuteri* CML393 and *B. velezensis* CML396 reduced inflammation and increased the expression of glucose transporter and the jejunum villus height, which might jointly contribute to the better chicken growth performance. As for the calcium signaling pathway, the p38 MAPK pathway affects calcium signal regulating lipid accumulation by changing PPARγ mRNA expression (44). In addition, calcium signal is closely related to muscle development, maintenance, and regeneration (45). Overall, our results confirmed the important role of the PPAR signaling pathway in modulating chicken physiology and metabolism and demonstrated that this pathway can be regulated by HBW-enriched beneficial bacteria, thus promoting chicken growth. However, as we showed that there were complex associations among gut microbes, SCMs, and DEGs, the mechanisms underlying the regulation processes still need to be elucidated. Based on the correlation analyses, we tentatively deduce that the lipid and lipid-like metabolites may be important mediators. This may be further supported by our observations that the expression of PDK4 was significantly increased in both the HBW and the consortium-treated chickens. The upregulation of PDK4 can modulate metabolic flux by promoting fatty acid metabolism in different tissues, and the PPAR signaling pathway is necessarily involved in upregulating PDK4 expression (46).

Although FMT is regarded as an effective manner to reconstruct gut microbiota and to transfer desired phonotypes from the donors to the receivers, our results showed that the transplantation of fecal microbiota from the HBW chickens to newborn chicks slightly affected chicken growth performance and gut core microbiome membership. This is consistent with the previous findings that fecal microbiota from highly feed-efficient chickens had no remarkable effects on the growth performance of newborn chicks in later life (7, 8). Similar results were also found in pigs that using inocula from highly feed-efficient pigs even negatively impacted the intestinal morphology and reduced body weight of the offspring (47). The reason for this may be due to that animals in their very early life cannot sustain or tolerate the complex microbes from the adults. Alternatively, the growth-promoting microbes, if exist, may fail to find their ecological niche when transferred together with other commensals to a new gut environment. Nonetheless, we showed that early-life FMT using the HBW chicken fecal microbiota enhanced the ability of antioxidant status, sugar transport, and immunity of the receivers, demonstrating the beneficial impact of early-life intervention of the chicken microbiota on the chicken phenotype.

Our results verified that administration of the combination of *L. reuteri* CML393 and *B. velezensis* CML396 isolated from the HBW chickens effectively improved the chicken growth performance, supporting the results of multi-omics analyses that *Lactobacillus* and *Bacillus* in the HBW chickens might be the key taxa in regulating the chicken physiology and metabolism and in contributing to the chicken growth and development. Very interestingly, among the six consortiums we tested, only the CML393 + CML396 combination had growth-promoting effects. We demonstrated in the following *in vitro* and *in silico* analyses that these two strains were less competitive but more cooperative in especially amino acid and fatty acid biosynthesis. These results highlight that (i) not all species in the genera of *Lactobacillus* and *Bacillus* in the HBW chickens are beneficial for chicken growth, and (ii) the interactions between specific bacterial species in these two genera are more important in exerting the growth-promoting effects on chickens. Based on the competitive exclusion principle (48), there is less competition stress among species in favor of their occupation of different niches, resulting in better synergetic effects for the members involved. More cooperation among members in a microbial consortium, for example, through metabolite cross-feeding, guarantees a more stable and enhanced output of a designed community (49). Although probiotic mixtures were found to bear better efficacy than single strain in health promotion and gut microbiota modulation (50), few efforts were made to characterize the bacterial interactions in a bacterial consortium. Our results, therefore, shed lights on how to select bacterial species or strains to construct a consortium used in both humans and animals.

In conclusion, we provide a comprehensive view of the different profiles of gut microbiota, gut content metabolites, host gene expressions, and their interactions in chickens from the same line differ in their body weight. We propose that the chicken cecal microbes, especially *Lactobacillus* and *Bacillus,* have considerable effects on modulating chicken gut function and resulting in better growth performance through microbial metabolites, possibly the lipids and lipid-like molecules and their crosstalk with the gut PPAR signaling pathways. Meanwhile, our study implies that the consortium consisting of *L. reuteri* CML393 and *B. velezensis* CML396 from the HBW chickens has the potential to be used as feed additives in improving chicken growth and development.

## MATERIALS AND METHODS

### Birds, experimental design, and sample collection

The feeding experiment was conducted with 150 newborn Arbor Acres chicks for a total period of 35 days. Corn-soybean meal diets with different nutritional levels were offered to all chickens from 0 to 21 days old and 22 to 35 days old, respectively. Chickens were kept in cages, and water was supplied to them with a nipple drinker. Diets were provided twice daily to ensure that chickens were free to eat and drink. Other management measures were carried out in accordance with conventional methods. Based on the body weight of chickens at 35 days old, 20 chickens with HBW and 20 chickens with LBW were selected. Chickens were weighed after 12 hours of fasting and then sacrificed under electric shock anesthesia. Ileal contents, cecal contents, and cecal tissue samples were placed in sterile centrifuge tubes and immediately frozen in liquid nitrogen containers. All samples were transferred and stored at −80°C in the laboratory until analysis. Contents of the ileum and cecum were utilized for microbiome analysis, and cecal contents were also retained for metabolome analysis. Cecal tissue samples were used for transcriptome analysis. All chickens were healthy and had not taken any antibiotics, probiotics, or prebiotics during the experimental period.

### Preparation for fecal microbiota suspension

In order to investigate the effect of microbiota on the growth performance and gut health of chickens, fresh feces were collected from the HBW and LBW chickens for FMT. The fecal microbiota suspension was prepared as described by Pang et al. under anaerobic conditions using an anaerobic system (51). First, the feces from each group were pooled, homogenized, and diluted with twofold sterile saline. Subsequently, the homogenate was filtered through 10-mesh, 18-mesh, 35-mesh, and 60-mesh sieves. Then, the filtrate was centrifuged at 6,000 × *g* for 15 min, and the precipitate was resuspended to a stock concentration of 1  ×  10^9^  CFU/mL in sterile saline with glycerol (20%, vol/vol) before gavaging.

### Microbiota transplantation

One hundred and eighty newborn Arbor Acres chicks with similar body weight were randomly divided into three groups with 10 replicates per group and received sterile saline or FMT for 14 days. A total of 200 µL of liquid was administered per chicken via oral gavage. The treatment groups were as follows: (i) Oral-CON group (*n* = 60), gavage of sterile saline; (ii) Oral-HBW group (*n* = 60), gavage of feces from HBW chickens; and (iii) Oral-LBW group (*n* = 60), gavage of feces from LBW chickens. Chickens were weighed and sacrificed under electric shock anesthesia on days 21 and 42. Serum was prepared from the blood centrifuged at 3,000 × *g* for 10 min at 4°C and frozen at −20°C for detecting antioxidant indicators. Jejunal tissue samples were used to measure the relative mRNA expression of sugar transporter, tight junction proteins, and inflammatory factors. Cecal contents were utilized for microbiome analysis.

### Targeted culturomics

The collected cecal contents of HBW chickens were opened and processed under a super-clean bench. Then, cecal content samples were serially diluted in sterile saline (1 g of content was squeezed into 9 mL sterile saline) and spread on MRS agar plates containing 0.5% (wt/vol) CaCO_3_ (BDH, Poole, United Kingdom) or LB agar plates. All plates were incubated at 37°C for 1–5 days. Well-separated colonies of different morphology were picked from each agar plate and purified by repeated streaking onto new plates. Each isolate was subjected to 16S rRNA gene amplicon sequencing analysis, and the amplification primers were 27F (5′-AGAGTTTGATCCTGGCTCAG-3′) and 1492R (5′-ACGGCTACCTTGTTACGACTT-3′). Using the taxonomically united database in EzBioCloud (http://www.ezbiocloud.net) (52), the full 16S rRNA gene nucleotide was definitively identified with high similarity. Then all isolates identified as *Lactobacillus* or *Bacillus* were resuspended in the corresponding growth medium with glycerol (20%, vol/vol) and stored at −80°C.

### Inoculation of bacterial strain

*Lactobacillus* (*Ligilactobacillus salivarius* CML391 and *Limosilactobacillus reuteri* CML393) and *Bacillus* (*Bacillus velezensis* CML396 and *Bacillus paralicheniformis* CML399) were obtained from cecal contents of the HBW chickens. Subsequently, they were cultured in MRS medium or LB medium for 12 hours at 37°C and then centrifuged at 5,000 × *g* for 5 min. The precipitate was mixed with skim milk (15%, wt/vol) and trehalose (5%, wt/vol) and immediately freeze-dried. The freeze-dried powder containing 10^11^–10^12^ colony-forming units (CFU)/g was stored in ziplock bags at 4°C until use. Four hundred ninety newborn Arbor Acres chicks with similar body weights were randomly divided into seven groups with seven replicates per group. The treatment groups were as follows: (i) CON group (*n* = 70), in which chickens were fed the basic diet; (ii) CML391 + CML396 group (*n* = 70), in which chickens were fed the basic diet supplemented with *L. salivarius* CML391 (10^9^ CFU/kg) and *B. velezensis* CML396 (10^9^ CFU/kg); (iii) CML391 + CML399 group (*n* = 70), in which chickens were fed the basic diet supplemented with *L. salivarius* CML391 (10^9^ CFU/kg) and *B. paralicheniformis* CML399 (10^9^ CFU/kg); (iv) CML393 + CML396 group (*n* = 70), in which chickens were fed the basic diet supplemented with *L. reuteri* CML393 (10^9^ CFU/kg) and *B. velezensis* CML396 (10^9^ CFU/kg); (v) CML393 + CML399 group (*n* = 70), in which chickens were fed the basic diet supplemented with *L. reuteri* CML393 (10^9^ CFU/kg) and *B. paralicheniformis* CML399 (10^9^ CFU/kg); (vi) CML391 + CML393 group (*n* = 70), in which chickens were fed the basic diet supplemented with *L. salivarius* CML391 (10^9^ CFU/kg) and *L. reuteri* CML393 (10^9^ CFU/kg); and (vii) CML396 + CML399 group (*n* = 70), in which chickens were fed the basic diet supplemented with *B. velezensis* CML396 (10^9^ CFU/kg) and *B. paralicheniformis* CML399 (10^9^ CFU/kg). Chickens were weighed and sacrificed under electric shock anesthesia on days 21 and 42. A 5 mm segment of the middle of the jejunum was fixed in 4% paraformaldehyde for sectioning and staining. Cecal contents were utilized for quantifying SCFAs. The sampling method of other sites was the same as above.

### DNA extraction, 16S rRNA gene sequencing, and microbiome analysis

Bacterial DNA was extracted from the ileal contents or cecal contents of chickens with a QIAamp DNA Stool Mini Kit (Qiagen, Germany) according to the manufacturer’s instructions. The library quality was determined using 1% agarose gel electrophoresis and EPOCH2 Microplate Readers (BioTek). The V3–V4 region of the bacterial 16S rRNA gene was amplified by polymerase chain reaction (PCR) using the 341F (5′- CCTACGGGNBGCASCAG-3′) and 805R (5′- GACTACNVGGGTATCTAATCC-3′) primer pair (53). The paired-end sequencing was performed using the Illumina Hiseq PE250 platform by the Institute of Microbiology of the Chinese Academy of Sciences (Beijing, China). The raw data obtained by sequencing then underwent quality filtering, denoising, merging, and amplicon sequence variants (ASVs) calling using the DADA2 plugin in QIIME2 (v2019.7) (54, 55). The ASVs were generated by clustering sequences with 100% similarity. Each ASV was assigned taxonomic information with a Naive Bayes classifier based on the SILVA database (56). Subsequently, bioinformatic analysis was performed based on this output normalized data.

The α-diversity of the microbiota was calculated using the R package vegan (57). The Bray-Curtis distance was used to conduct principal coordinate analysis in order to evaluate the overall difference in microbiota structure between the HBW and LBW groups. In addition, the two groups were statistically compared using PERMANOVA and PERMDISP to evaluate the rationality of the group divisions. Differentially abundant taxa were discovered by LEfSe with the parameter “LDA score > 2.” RF analysis was a machine learning method that attempted to find the combination of metrics that most accurately differentiated between the HBW and LBW groups. Meanwhile, ROC curve analysis based on the random forest models was also performed, and we calculated the AUC, sensitivity, specificity, and accuracy to evaluate the diagnostic efficacy of the model. The tools for RF and ROC were randomForest and riskRegression in R software, respectively. The co-occurrence networks were constructed using Spearman correlations, and only the genus-level bacterial taxa with a relative abundance > 0.1% and prevalence > 75% were used in the co-occurrence networks. To describe the topology of the network, several parameters (e.g., Degree, ClusteringCoefficient, AverageShortestPathLength, BetweennessCentrality, ClosenessCentrality, NeighborhoodConnectivity, Radiality, Stress, TopologicalCoefficient, and Eccentricity) were calculated (58).

### Cecal metabolite measurements and bioinformatics analysis

Cecal metabolites were measured by non-targeted metabolomic profiling using the ultra performance liquid chromatography-mass spectrometry system (LC-MS, Agilent Technologies, Atlanta, GA, USA). The processing methods of cecal contents were done in the following way: 100 mg of cecal contents was homogenized in a 2 mL centrifuge tube. Four hundred microliters of precooled methanol/acetonitrile (1:1, vol/vol) was added, vortexed for 30 s, kept in an ultrasonic water bath for 10 min at 4°C, and left to stand overnight at −80°C. The cecal contents were taken out from the refrigerator at −80°C and thawed at 4°C. After thawing, they were centrifuged at 12,000 × *g* for 15 min at 4°C. The supernatant was dried by vacuum centrifugation (CV200; Beijing Gem Technology Co., Ltd., Beijing, China). Then, 200 µL methanol/acetonitrile (1:1, vol/vol) was added, kept in an ultrasonic water bath for 10 min at 4°C, and centrifuged at 12,000 × *g* for 15 min at 4°C. The supernatant was placed in vials and stored at −80°C until analysis. Each 2.0 µL sample was injected into an Agilent ZORBOX Eclipse Plus C18 column (100 × 2.1 mm i.d., 1.8 µm) whose temperature was set to 40°C by LC-MS. The mobile phase was a mixture of 0.1% formic acid-water (A) and 0.1% formic acid-acetonitrile (B) at a flow rate of 0.3 mL/min. The gradient elution program was as follows: 0–2 min, 5% (B); 2–20 min, 5%–100% (B); 20–25 min, 100% (B). Under mass spectrometry conditions, the ion source parameters of 325°C, drying gas flow of 8 L/min, nebulizer of 35  psi, sheath gas heater of 360°C, sheath gas flow of 12  L/min, a capillary voltage of 4.0 kV for positive mode, and 3.5 kV for negative mode.

The obtained LC-MS data were further analyzed by Agilent Profinder software for retention time correction, peak identification, peak extraction, peak integration, and peak alignment. Metabolites were identified by the Human Metabolome Database (HMDB) (59). The metabolites with the same HMDB ID were integrated, and then the metabolites in the positive and negative ion modes were merged into a metabolite matrix. Wilcoxon rank-sum test of the metabolite matrix was conducted using the R software. Orthogonal partial least squares discriminant analysis was performed to visualize metabolic differences between the HBW and LBW groups using the SIMCA software (v14.1, Umetrics, Sweden). VIP value was extracted from the OPLS-DA model. SCMs were defined as VIP > 1 and adjusted *P*-value < 0.05. The category and functional enrichment of the SCMs were analyzed using the MetOrigin platform (60).

### RNA extraction and transcriptome analysis from cecal samples

For cecal tissue transcriptomic analysis, the total RNA of each sample was extracted using TRIzol reagent (Invitrogen, USA) according to the manufacturer’s instructions. RNA was quantified and qualified by 1% agarose gel and EPOCH2 Microplate Readers (BioTek). The complementary DNA (cDNA) libraries were constructed following Illumina standard protocols and sequenced with HiSeq-PE150. The construction of cDNA libraries can be divided into six steps. Ribosomal RNAs were removed from total RNA samples, and then the RNA was divided into fragments and converted to cDNA. Double-stranded cDNA was synthesized using deoxyadenosine triphosphate, deoxycytidine triphosphate, deoxythymidine triphosphate, and deoxyguanosine triphosphate. Afterward, adaptors were ligated to the ends of these 3′ adenylated cDNA fragments, and PCR amplification followed. The construction and sequencing of the cDNA library were performed by the Institute of Microbiology of the Chinese Academy of Sciences (Beijing, China).

To obtain high-quality clean reads, the raw reads were further filtered by removing reads containing adapters, reads containing poly-N, and reads with low quality. Subsequently, Q20, Q30, and GC content of clean reads were calculated by FastQC software (61). The reference genome of chickens (Gallus_gallus.GRCg6a.dna.toplevel.fa.gz) and annotation files (Gallus_gallus.GRCg6a.102.gtf) were downloaded from the Ensembl database (http://www.ensembl.org/index.html). An index of the reference genome of chickens was built, and then we aligned paired-end clean reads to the reference genome using Hisat2 (v2.1.0) (62). At the same time, the alignment files were saved in SAM format, which was then converted to BAM format files using Samtools (v1.12) (63). Mapped reads were assembled by StringTie (v1.12) to estimate the expression level of the gene or transcript (64). Differentially expressed genes were calculated with DeSeq2 package in R software (65), and the screening threshold was |log2(fold change)|  > 1 and adjusted *P*-value < 0.05. Subsequently, KEGG pathway enrichments were performed for DEGs using the KOBAS 3.0.

### Intestinal morphology analysis

For morphological measurements, 4% paraformaldehyde-fixed jejunum samples were embedded in paraffin and stained with hematoxylin and eosin. Images of five visual fields (one section per chicken) were randomly taken under the microscope (JS-500 binocular biological microscope; LIOO, Beijing, China) at 40× magnification. Then, the images were measured, including villus height and crypt depth.

### Determination of cecal content SCFAs

The concentrations of SCFAs from cecal contents were measured using gas chromatography (66). Cecal contents were diluted with double-distilled water (fourfold volume of the content) and centrifuged at 12,000 × *g* for 15 min at 4°C. About 0.2 mL of freshly prepared 25% metaphosphoric acid was added to 1 mL of supernatant, mixed well, and left to stand for 30 min at 4°C. The mixture was then centrifuged at 12,000 × *g* for 10 min at 4°C, and the supernatant was stored at −20°C prior to the determination of the concentration of total SCFAs, acetate, propionate, butyrate, isobutyrate, valerate, and isovalerate.

### Real-time quantitative PCR

After extracting the total RNA of jejunal tissue samples, the first-strand cDNA was synthesized by reverse transcription using BeyoRT II First Strand cDNA Synthesis kit with gDNA Eraser (Beyotime Institute of Biotechnology). The RT-qPCR reactions were conducted in QuantStudio 7 Flex (Applied Biosystems, USA) equipment. The samples in each group had three complex holes, and the specificities of RT-qPCR products were verified by agarose gel electrophoresis followed by melting curve analysis. The relative expression level of each mRNA normalized to glyceraldehyde-3-phosphate dehydrogenase was calculated by the 2^−ΔΔCt^ method (67). All primers were designed in exon-exon junctions using the Primer-BLAST tool (https://www.ncbi.nlm.nih.gov/tools/primer-blast/). The primers used were synthesized by Sangon Biotech (Shanghai, China) and listed in Table S13.

### Assessment of serum antioxidant capacity

Serum antioxidant capacity, such as the T-AOC, CAT, T-SOD, GSH-PX, and MDA in serum samples, was measured by commercially available kits (Nanjing Jiancheng Institute of Bioengineering, Nanjing, Jiangsu, China), according to the manufacturer’s instructions.

### Whole-genome sequencing

*L. salivarius* CML391, *L. reuteri* CML393, *B. velezensis* CML396, and *B. paralicheniformis* CML399 were sequenced using the Illumina Hiseq 2500 platform by the Institute of Microbiology of Chinese Academy of Sciences (Beijing, China). Fastp and FastQC were employed for quality filtering of raw reads and quality control, respectively. Clean reads were then assembled into contiguous sequences (contigs) using SPAdes (v3.1.0) (68), and their quality was evaluated with QUAST (v4.6.3) (69). For draft genomes, Prokka automatic annotation software was used to annotate the genomes, including coding sequences, tRNA, rRNA, etc. (70). Proksee (https://proksee.ca) was applied to draw a genome map based on the annotation results. The genome sequences of the four strains and the corresponding species were searched and downloaded from the NCBI Genome database (https://www.ncbi.nlm.nih.gov/data-hub/genome/). Single nucleotide polymorphisms were identified using kSNP3 software (71), and then a phylogenetic tree was constructed based on Maximum Likelihood using the iTOL (https://itol.embl.de/). Additionally, we predicted genome-scale metabolic potentials using gapseq (72), and gapseq output was screened for a hand-curated set of pathways by using MetaCyc pathways (73).

### Generation of spent culture media and growth measurements

Bacterial spent culture supernatants were obtained by centrifugation of the densely grown subculture at 4°C for 20 min at 5,000 × *g* and filter sterilization of 0.22 µm. Growth of monoculture in the MIX medium (MRS and LB medium were mixed at 1:1 ratio) or SM medium of other strains was then measured in 96-well round bottom plates (Nunc) using a microplate reader. The reaction volume was 100 µL, and the initial inoculum concentration remained consistent. During continuous measurements, an absorbance reading at λ = 600 nm was taken every 2 h pre-time for 48 h, and the plate was heated inside the reader at 37°C and a 30 s double orbital shaking step was performed prior to every measurement.

### Statistical analysis and figures

Statistical analysis was performed using GraphPad Prism version 8.0 (San Diego, CA, USA), and all data were represented as means  ±  standard error. We applied the student’s *t*-test or Wilcoxon rank test to verify the statistical significance between the two groups. Data sets involving more than two groups were evaluated by one-way analysis of variance followed by least significant difference or by the non-parametric Kruskal-Wallis test with IBM SPSS Statistics 24.0 (SPSS Inc., Chicago, IL, USA). Meanwhile, the *P-*value was corrected using the Benjamini–Hochberg method to control the false discovery rate. Statistical significance was defined as *P*-value < 0.05, whereas *P-*values between 0.05 and 0.10 were considered as a trend. Figures were partly generated using BioRender (https://biorender.com) and Adobe Illustrator CC (Adobe Inc.).

## Data Availability

All sequencing data and assembled genomes have been deposited in the NCBI Sequence Read Archive (SRA) database under the accession numbers PRJNA913411 (microbiota raw sequence data), PRJNA913464 (RNA-seq raw data), and PRJNA913094 (assembled genomes). All other relevant data are available in the main paper and supplemental files.

## SUPPLEMENTAL MATERIAL

Supplemental figures msystems.00844-23-s0001.pdf Fig. S1 to S8.

Supplemental tables msystems.00844-23-s0002.xlsx Tables S1 to S13.

ASM does not own the copyrights to Supplemental Material that may be linked to, or accessed through, an article. The authors have granted ASM a non-exclusive, world-wide license to publish the Supplemental Material files. Please contact the corresponding author directly for reuse.
