# Supplementary material for: Integrated multi-omics reveals the roles of cecal microbiota and its derived bacterial consortium in promoting chicken growth
Source: mSystems. 2023 Nov 29;8(6):e00844-23. doi: 10.1128/msystems.00844-23 (PMC10734529; doi:10.1128/msystems.00844-23)
Supplement: Supplemental figures — Fig. S1 to S8. [file msystems.00844-23-s0001.pdf]

Supplementary Figures for

Integrated multi-omics reveals the roles of cecal microbiota and its derived bacterial consortium in promoting chicken growth

Meihong Zhang,[a] Depeng Li,[a] Xinyue Yang,[a] Fuxiao Wei,[a] Qiu Wen,[a] Yuqing Feng,[a] Xiaolu Jin,[a] Dan Liu,[a] Yuming Guo,[a] Yongfei Hu[a]#

[a]State Key Laboratory of Animal Nutrition and Feeding, College of Animal Science and Technology, China Agricultural University, Beijing 100193, China.

#Address correspondence to Yongfei Hu, huyongfei@cau.edu.cn

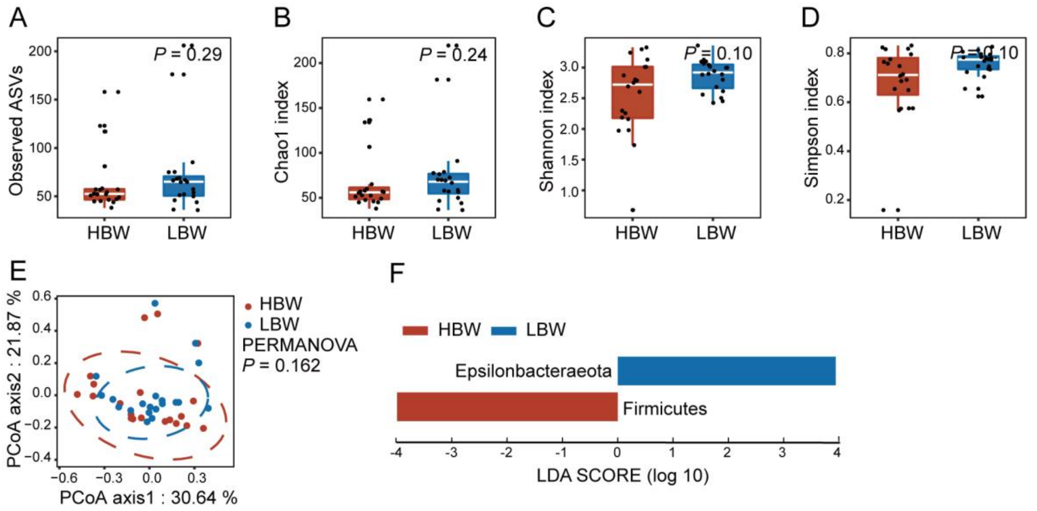

Fig. S1 Differences of ileal microbiota composition in the HBW and LBW chickens. Observed ASVs **(A)**, Chao1 index **(B)**, Shannon index **(C)** and Simpson index **(D)** between the HBW and LBW chickens. **(E)** Principal coordinate analysis (PCoA) plot based on Bray-Curtis distance of microbiota composition between the HBW and LBW chickens. **(F)** Differentially abundant taxa were tested by linear discriminant analysis effect size (LEfSe), with linear discriminant analysis (LDA) score > 2 and *P-value* < 0.05.

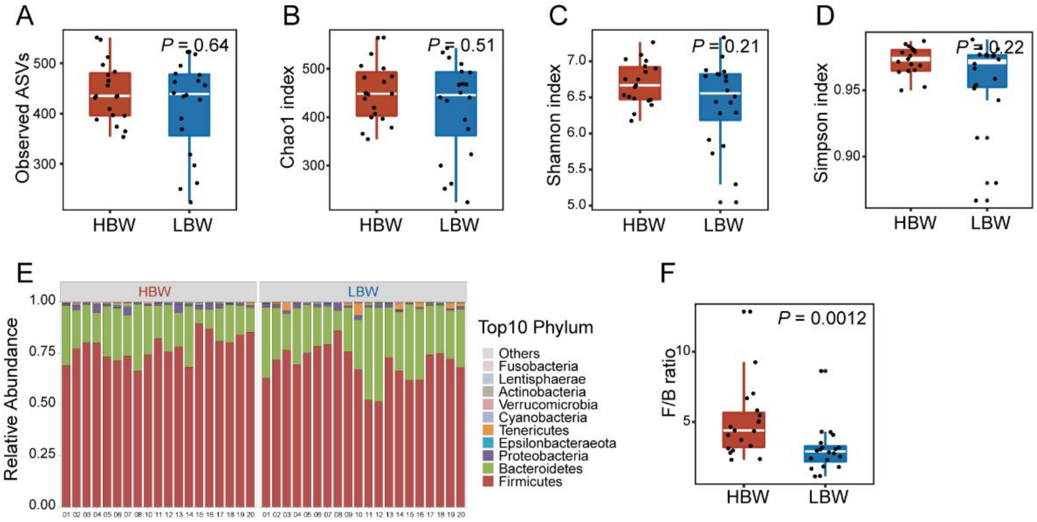

Fig. S2 Differences of cecal microbiota composition in the HBW and LBW chickens. Observed ASVs **(A)**, Chao1 index **(B)**, Shannon index **(C)** and Simpson index **(D)** between the HBW and LBW chickens. **(E)** Relative abundances of cecal microbiota at the phylum level in the HBW and LBW chickens. **(F)** Firmicutes/Bacteroidetes ratio in the HBW and LBW chickens.

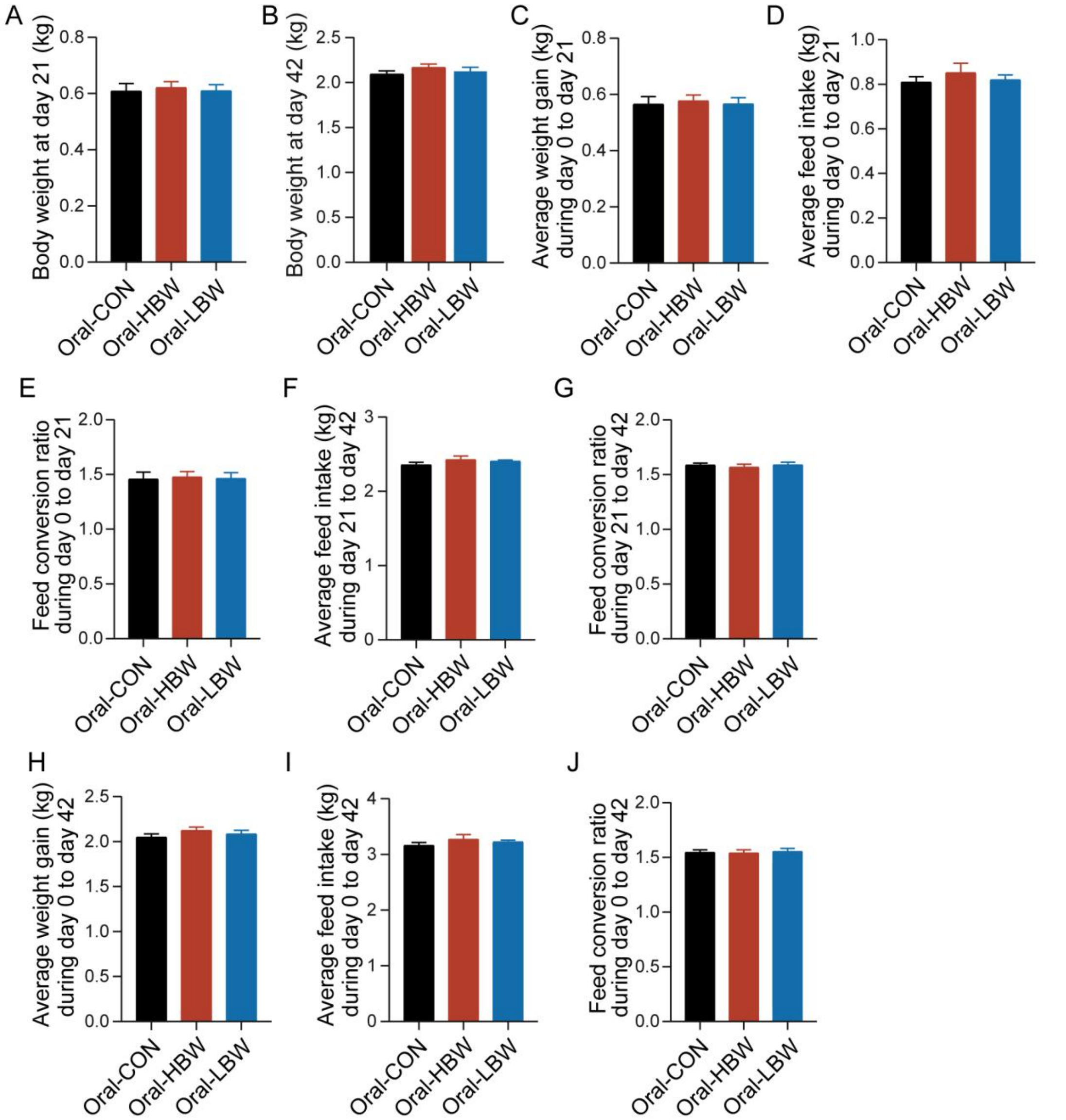

Fig. S3 Effects of FMT on chicken growth performance. **(A)** Body weight at the 21th day. **(B)** Body weight at the 42th day. Average weight gain **(C)**, average feed intake **(D)** and feed conversion ratio **(E)** during day 0 to day 21. Average feed intake **(F)** and feed conversion ratio **(G)** during day 21 to day 42. Average weight gain **(H)**, average feed intake **(I)** and feed conversion ratio **(J)** during day 0 to day 42.

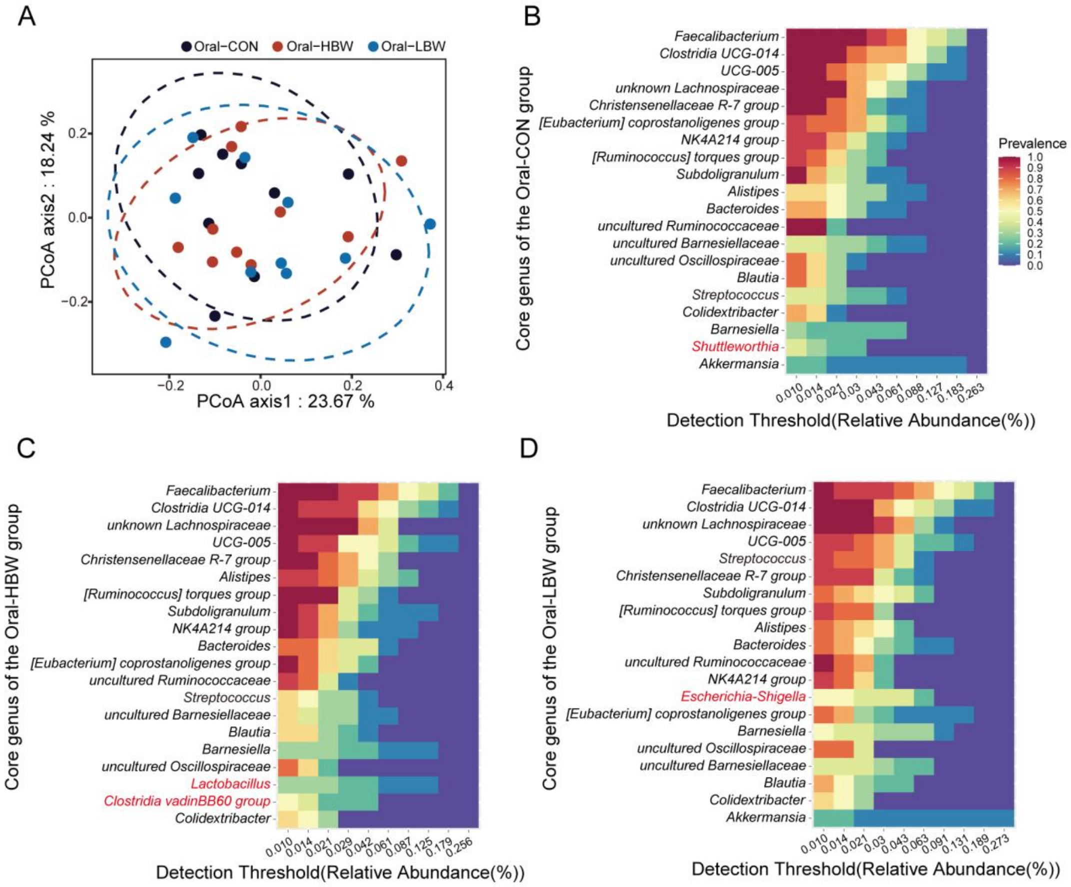

Fig. S4 Effects of FMT on chicken cecal microbiota. **(A)** PCoA plot based on Bray-Curtis distance of microbiota composition among the oral-CON, oral-HBW and oral-LBW chickens. The top20 core microbiome membership of the oral-CON **(B)**, oral-HBW **(C)** and oral-LBW **(D)** group.

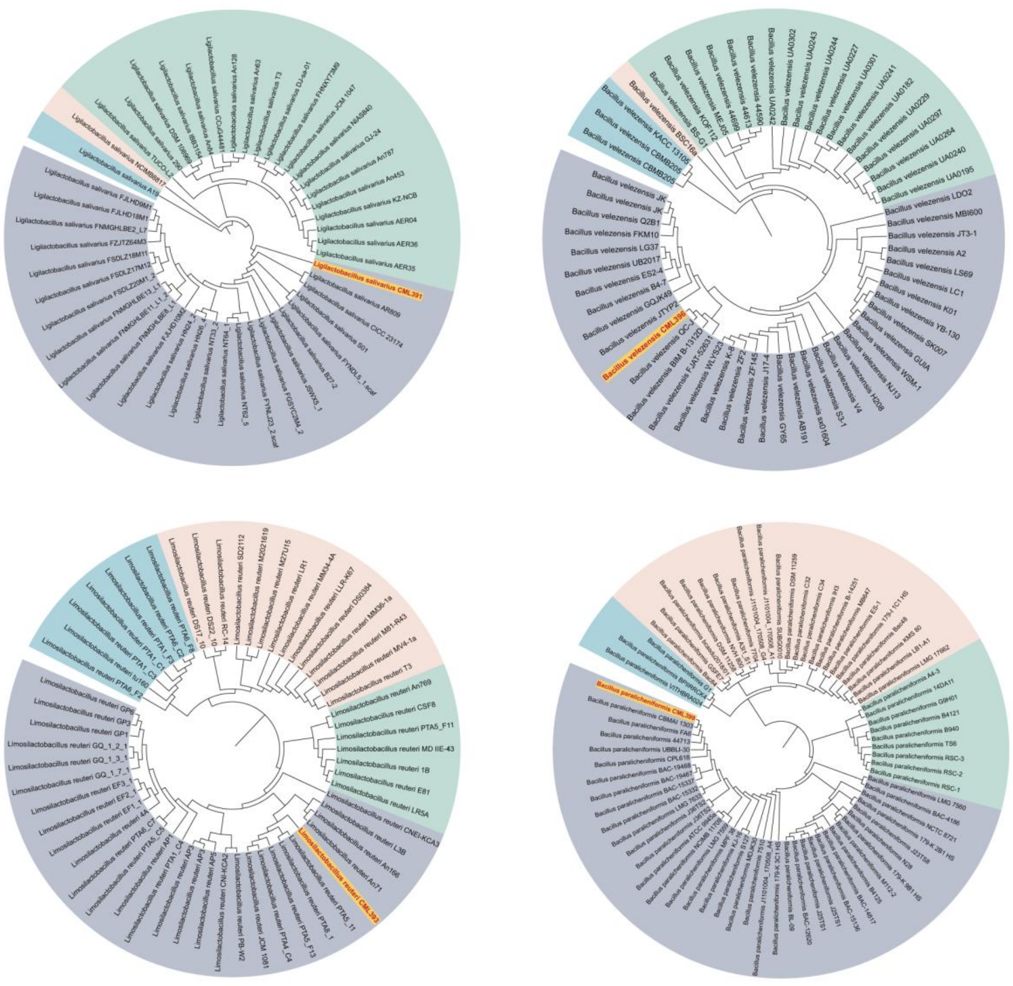

Fig. S5 The phylogenetic trees of *L. salivarius* CML391, *L. reuteri* CML393, *B. velezensis* CML396 and *B. paralicheniformis* CML399. Different colors represent different branches.

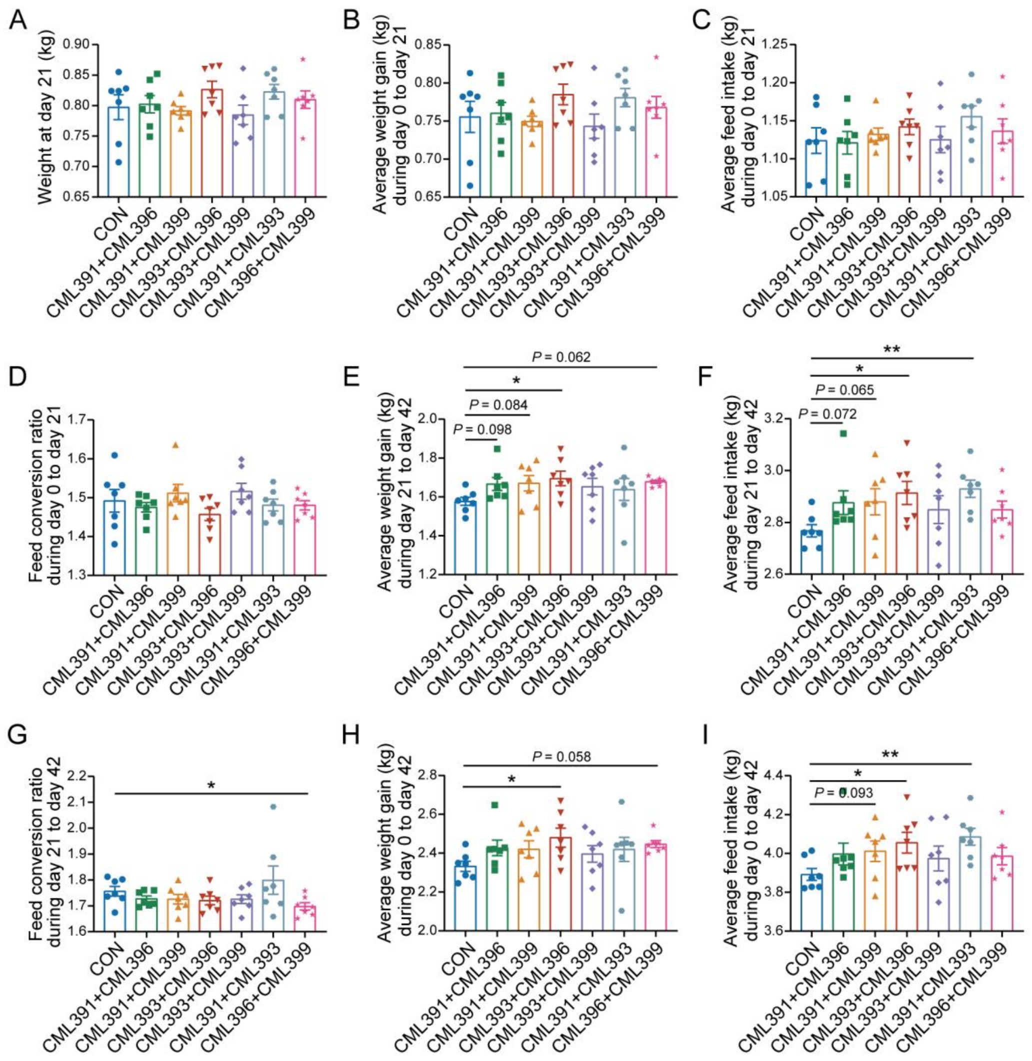

Fig. S6 Effects of dietary supplementation with *Lactobacillus* and *Bacillus* on chicken growth performance. **(A)** Body weight at the 21th day. Average weight gain **(B)**, average feed intake **(C)** and feed conversion ratio **(D)** during day 0 to day 21. Average weight gain **(E)**, average feed intake **(F)** and feed conversion ratio **(G)** during day 21 to day 42. Average weight gain **(H)** and average feed intake **(I)** during day 0 to day 42.

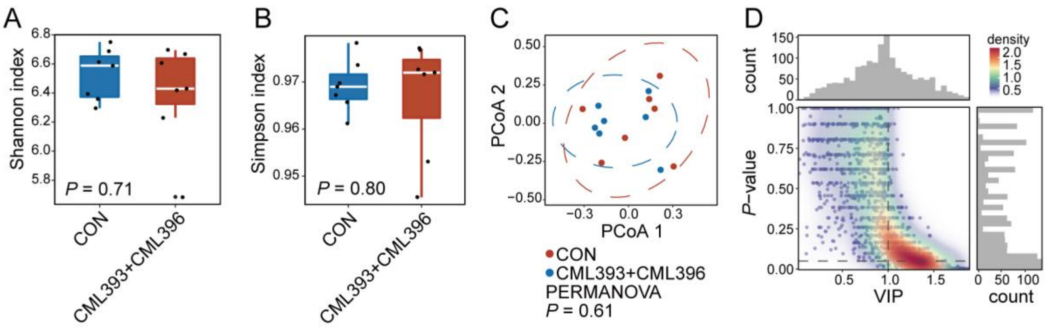

Fig. S7 Effects of dietary supplementation with *L. reuteri* CML393 and *B. velezensis* CML396 on cecal microbiota and metabolites of chickens. Shannon index **(A)** and Simpson index **(B)** between the CON and CML393+CML396 chickens. **(C)** PCoA plot based on Bray-Curtis distance of microbiota composition between the CON and CML393+CML396 chickens. **(D)** SCMs in the CON and CML393+CML396 chickens. Top panel: density of the *VIP* of the metabolites, right panel: density of the *P-value*.

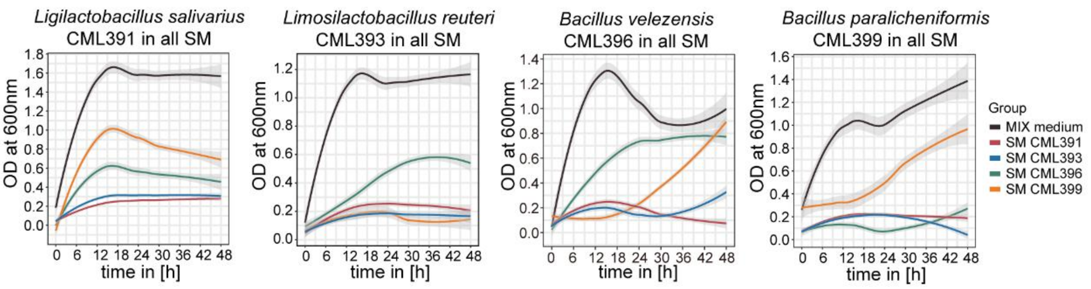

Fig. S8 Growth curves of individual strains in the MIX medium (MRS+LB) and the sterile spent culture medium (SM) of *L. salivarius* CML391, *L. reuteri* CML393, *B. velezensis* CML396 and *B. paralicheniformis* CML399. Growth of all individual monoculture was monitored in fresh MIX medium (black) and in SM of individual four strains (colored lines) over 48 hours at OD 600nm. The mean of three independent experiments is shown with the corresponding SD (grey).
